# Supplementary material for: Mechanistic insights into glycoside 3-oxidases involved in C-glycoside metabolism in soil microorganisms
Source: Nat Commun. 2023 Nov 14;14:7289. doi: 10.1038/s41467-023-42000-3 (PMC10646112; doi:10.1038/s41467-023-42000-3)
Supplement: Supplementary file 1 — Supplementary Information [file 41467_2023_42000_MOESM1_ESM.pdf]

Table of Contents

1. SUPPLEMENTARY FIGURES 2

SUPPLEMENTARY FIGURE 1........ 2
SUPPLEMENTARY FIGURE 2........ 3
SUPPLEMENTARY FIGURE 3........ 4
SUPPLEMENTARY FIGURE 4........ 5
SUPPLEMENTARY FIGURE 5........ 6
SUPPLEMENTARY FIGURE 6........ 7
SUPPLEMENTARY FIGURE 7........ 8
SUPPLEMENTARY FIGURE 8........ 9
SUPPLEMENTARY FIGURE 9........ 10
SUPPLEMENTARY FIGURE 10........ 11
SUPPLEMENTARY FIGURE 11........ 12
SUPPLEMENTARY FIGURE 12........ 13
SUPPLEMENTARY FIGURE 13........ 14
SUPPLEMENTARY FIGURE 14........ 15
SUPPLEMENTARY FIGURE 15........ 16
SUPPLEMENTARY FIGURE 16........ 17
SUPPLEMENTARY FIGURE 17........ 18
SUPPLEMENTARY FIGURE 18........ 19
SUPPLEMENTARY FIGURE 19........ 20
SUPPLEMENTARY FIGURE 20........ 21
SUPPLEMENTARY FIGURE 21........ 22
SUPPLEMENTARY FIGURE 22........ 23
SUPPLEMENTARY FIGURE 23........ 24
SUPPLEMENTARY FIGURE 24........ 25
SUPPLEMENTARY FIGURE 25........ 26
SUPPLEMENTARY FIGURE 26........ 27
SUPPLEMENTARY FIGURE 27........ 28
SUPPLEMENTARY FIGURE 28........ 29
SUPPLEMENTARY FIGURE 29........ 30
SUPPLEMENTARY FIGURE 30........ 31
SUPPLEMENTARY FIGURE 31........ 32
SUPPLEMENTARY FIGURE 32........ 33
SUPPLEMENTARY FIGURE 33........ 34
SUPPLEMENTARY FIGURE 34........ 35

2. SUPPLEMENTARY TABLES........ 36

SUPPLEMENTARY TABLE 1. 36
SUPPLEMENTARY TABLE 2. 37
SUPPLEMENTARY TABLE 3. 38
SUPPLEMENTARY TABLE 4. 39
SUPPLEMENTARY TABLE 5. 40
SUPPLEMENTARY TABLE 6. 41
SUPPLEMENTARY TABLE 7. 42
SUPPLEMENTARY TABLE 8. 43
SUPPLEMENTARY TABLE 9. 44
SUPPLEMENTARY TABLE 10. 45
SUPPLEMENTARY TABLE 11. 46
SUPPLEMENTARY TABLE 12 49

4. SUPPLEMENTARY REFERENCES........ 50

1. Supplementary Figures

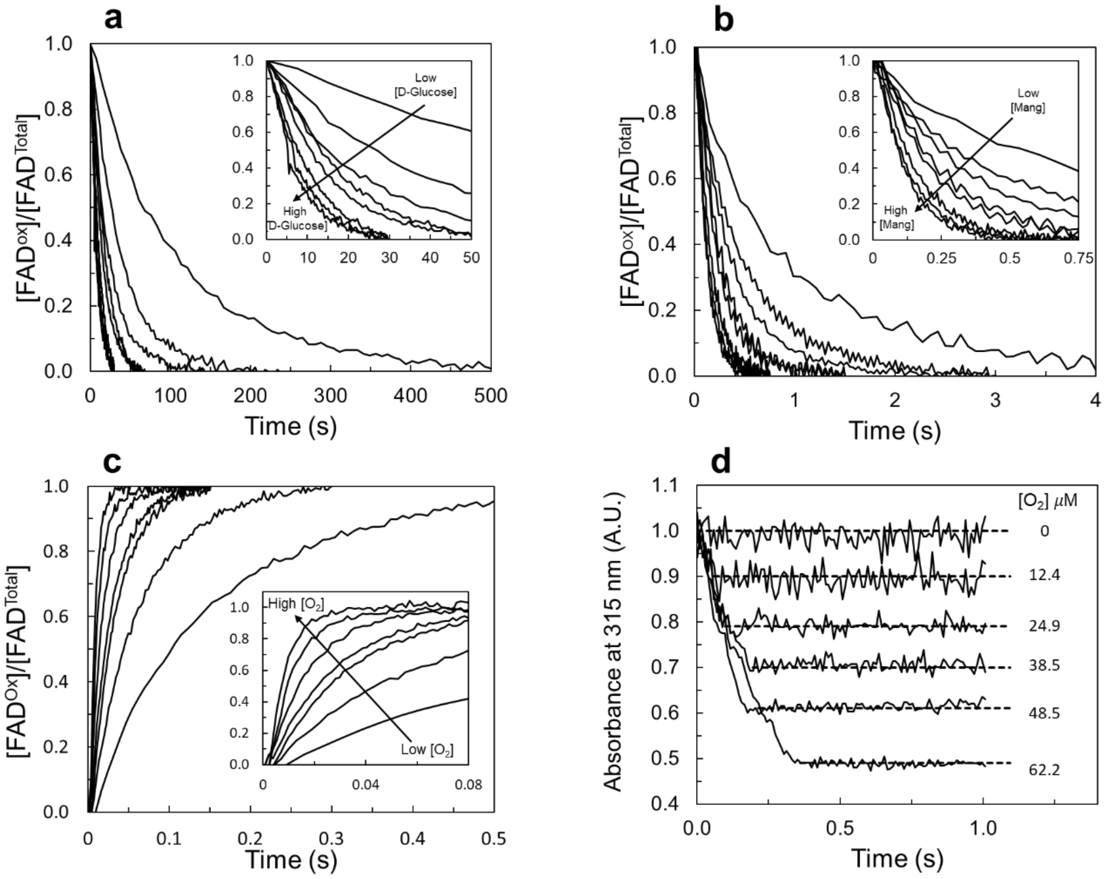

SUPPLEMENTARY FIGURE 1. Example of normalized raw data of transient state kinetics for the wild-type *Ps*G3Ox and dioxygen quantification in the samples. (a) Normalized traces were obtained for the reductive half-reaction using D-Glc as substrate and following the reduction of FAD. In the inset zooms, the traces were taken at shorter timescales. The final concentration of D-Glc was 0.05, 0.15, 0.25, 0.35, 0.45, 0.55, 0.65, and 1 M. **(b)** Normalized traces were obtained for the reductive half-reaction using Mang as substrate and following the reduction of FAD. The final concentration of Mang was 0.05, 0.075, 0.1, 0.15, 0.2. 0.25, 0.3 and 0.4 mM. **(c)** Normalized traces were obtained for oxidative half-reaction following the oxidation of FAD. The oxygen concentration in the samples used in the oxidative half-reaction was determined using sodium dithionite and monitoring the absorbance changes at 315, as shown in **(d)**. The horizontal lines represent the average absorbance at 315 nm to calculate the $\Delta Abs_{315,}$ allowing further determination of consumed dithionite, equivalent to O_2 present in the samples. All assays were performed under an anaerobic atmosphere at 25ºC in 100 mM sodium phosphate buffer, pH 7.5, with 200 mM NaCl. Source data are provided as a Source Data file.

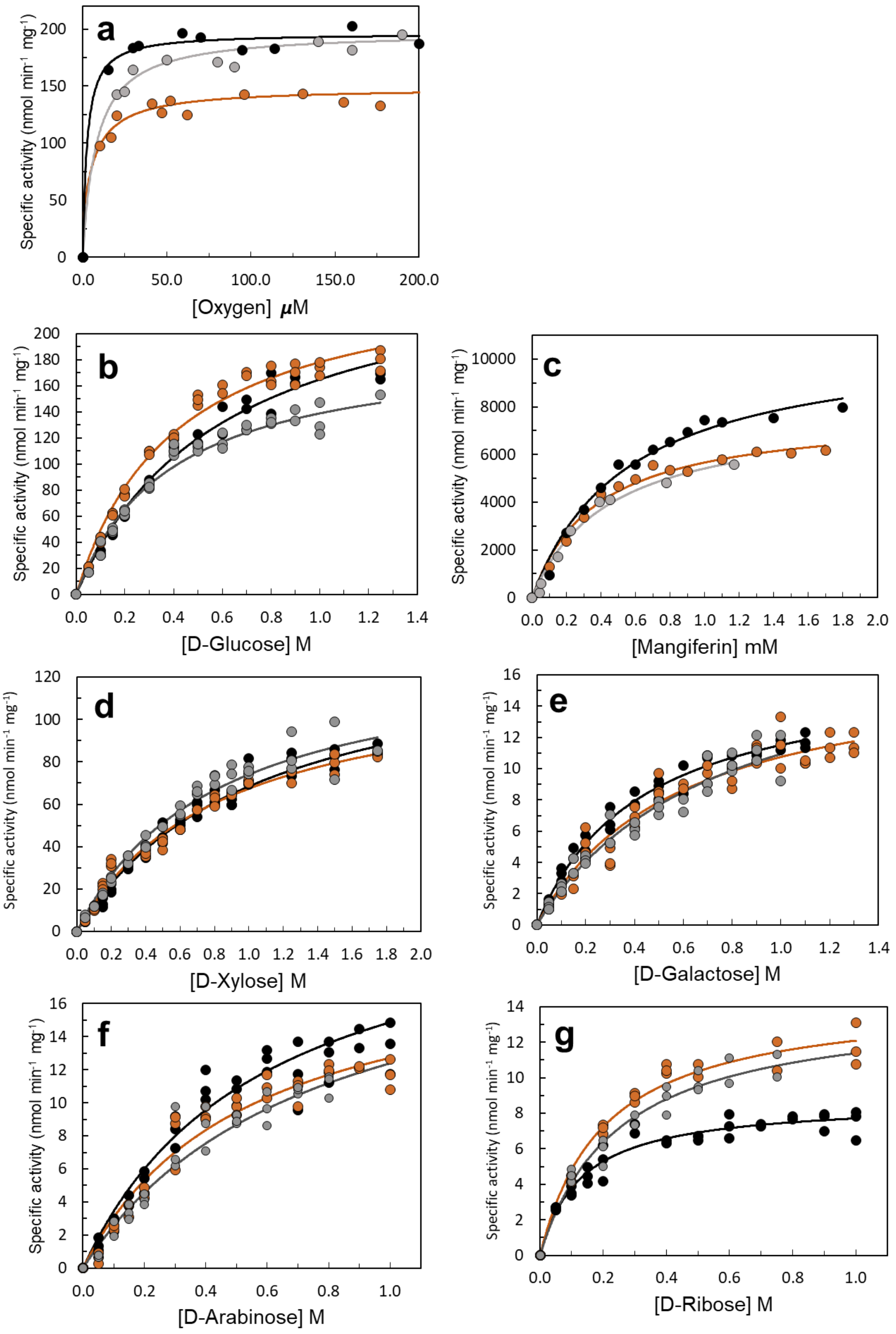

SUPPLEMENTARY FIGURE 2. Apparent steady-state kinetics of wild-type *Ps*G3Ox at 37 ºC in 100 mM sodium phosphate buffer at pH 7.5**.** The dots represent the experimental enzymatic activities measured, and the lines are the fit of the experimental data to the Michaelis-Menten equation ($v = \frac{V_{\mathrm{max}}[\mathrm{S}]}{\mathrm{K_m}+[\mathrm{S}]}$) using OriginLab software. The independent experiments (n=3) are represented in distinct colors. The activity for dioxygen was obtained by measuring the oxygen consumption in an Oxygraph using 1 M of D-Glc as the electron donor. The kinetics for Mangiferin was obtained by measuring the oxygen consumption in an Oxygraph. The oxidation rate of the monosaccharides was followed using the HRP-AAP/DCHBS coupled assay. Source data are provided as a Source Data file.

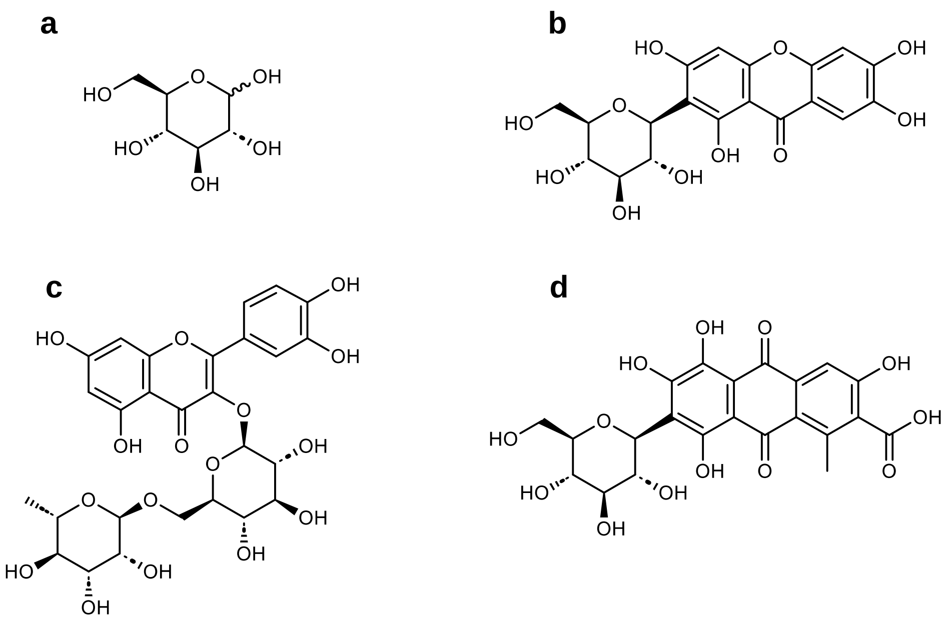

Supplementary Figure 3. Chemical structures of substrates used in this study. (a) D-Glucose (D-Glc), (b) Mangiferin (Mang), (c) Rutin and (d) Carminic acid.

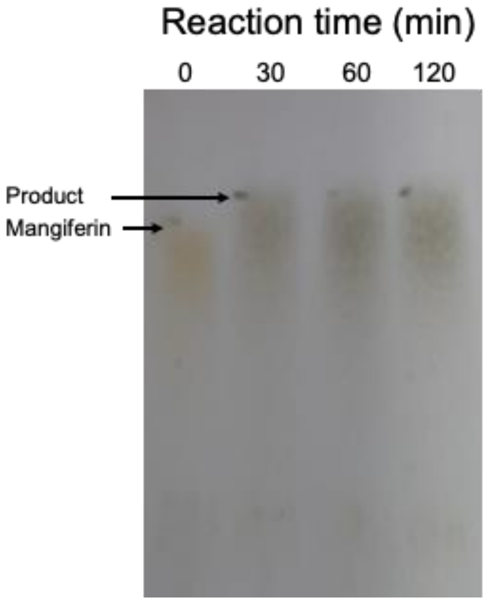

Supplementary Figure 4. TLC analysis of the time-course oxidation of Mang. Aliquots of the reaction mixture were taken before the addition of *Ps*G3Ox (0 min) and 30, 60, and 120 min after the addition of the enzyme. The TLC was performed in silica gel 60 F_{254} plates using a mixture of butanol, acetic acid, and water 4:1:2.2 (v/v) as the mobile phase. The TLC was revealed using a diphenylamine-aniline-phosphoric acid reagent[1].

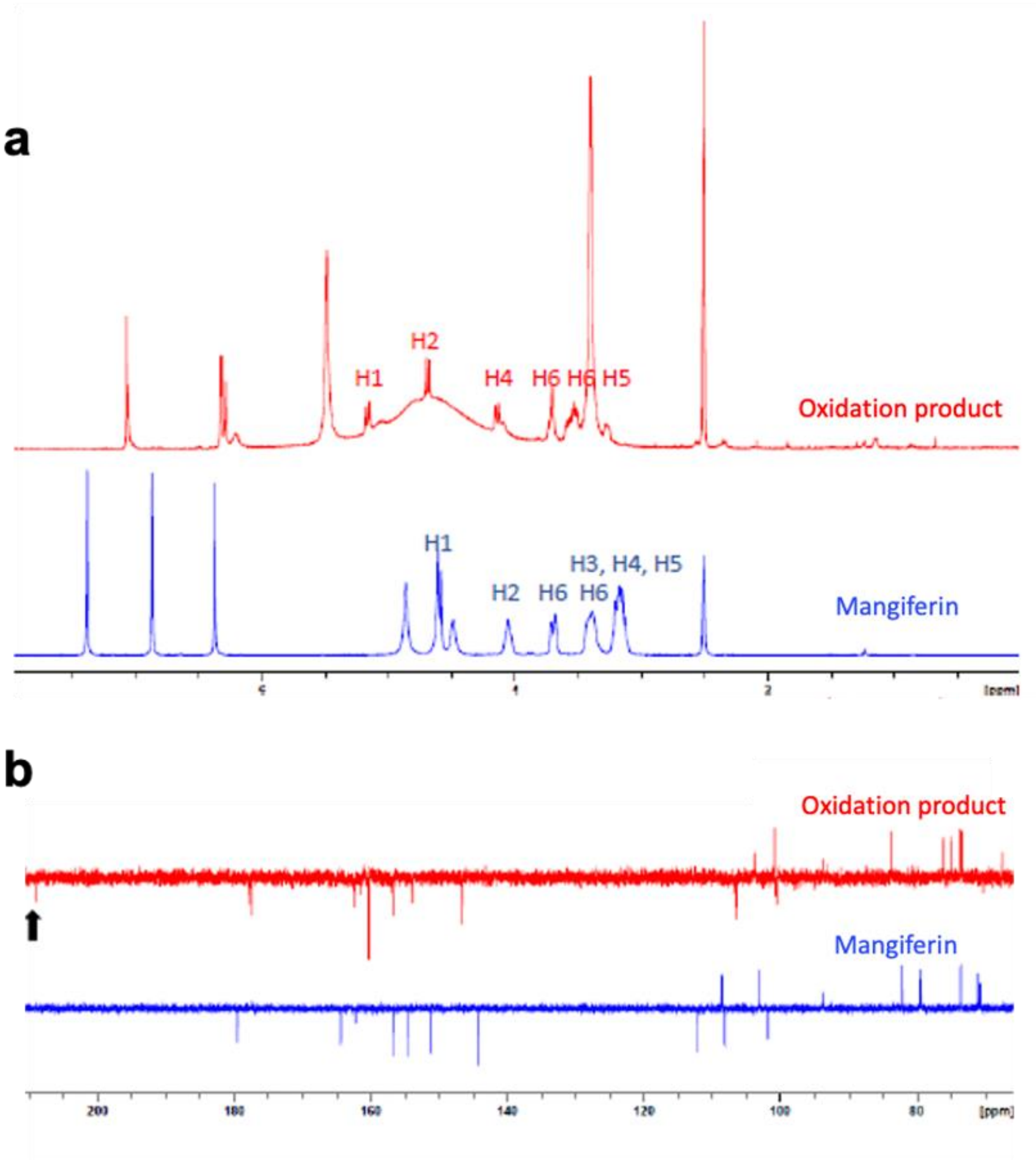

Supplementary Figure 5. Analysis of the Mang and the product of the reaction catalysed by *Ps*G3Ox. (a) ^{1}H NMR spectra of Mang (blue) and the product of its oxidation by *Ps*G3Ox (red) in DMSO-d_6, the protons of the sugar moiety are identified near the peaks. (b) ^{13}C APT NMR spectra of Mang (blue) and the product of its oxidation by *Ps*G3Ox (red) in DMSO-d_6, the black arrow highlights a new peak at 209 ppm which suggests a new ketone formed in the reaction catalysed by *Ps*G3Ox and that has been attributed to the C3.

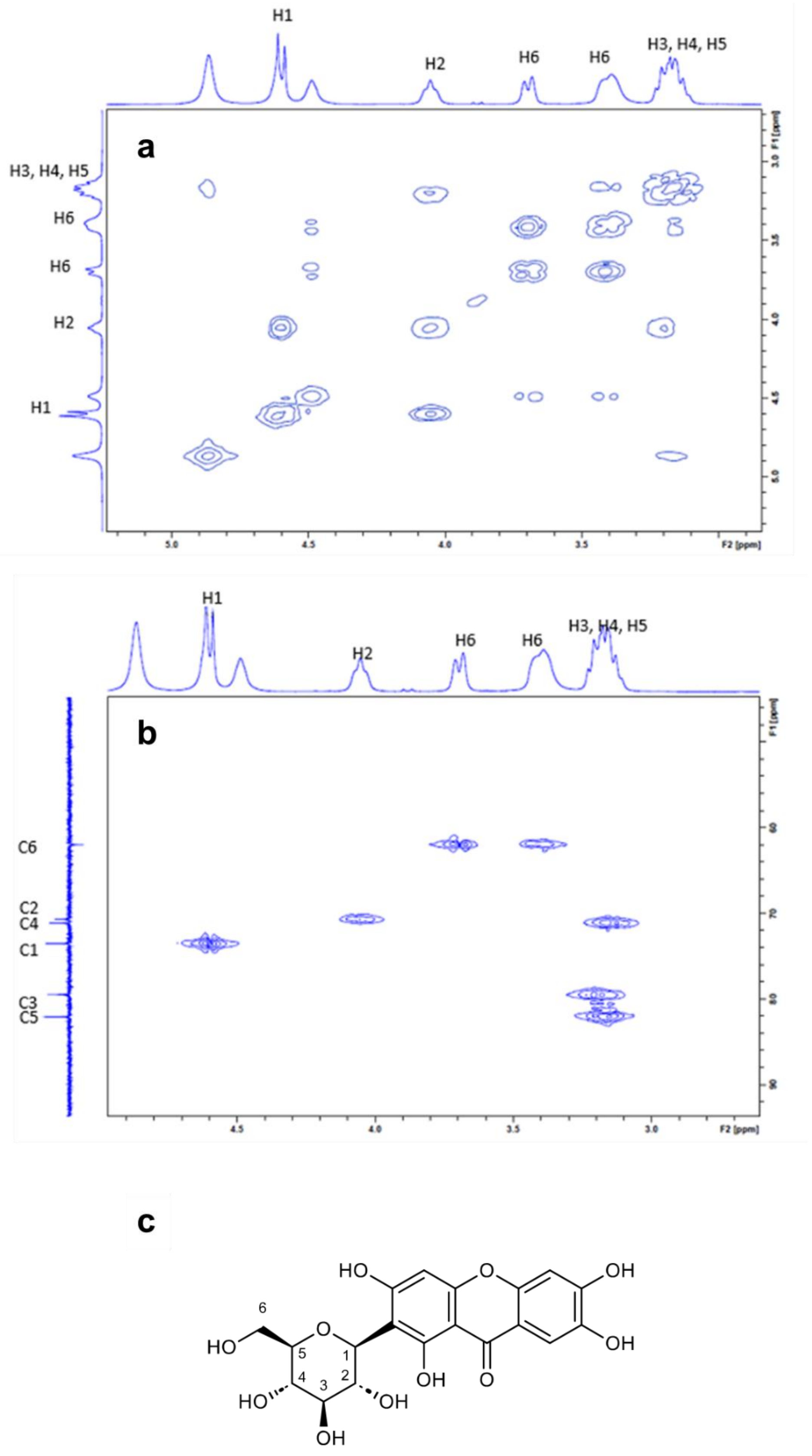

Supplementary Figure 6. NMR characterization of Mang. (a) 2D-COSY NMR spectrum. (b) HMQC NMR spectrum. (c) Chemical structure of Mang.

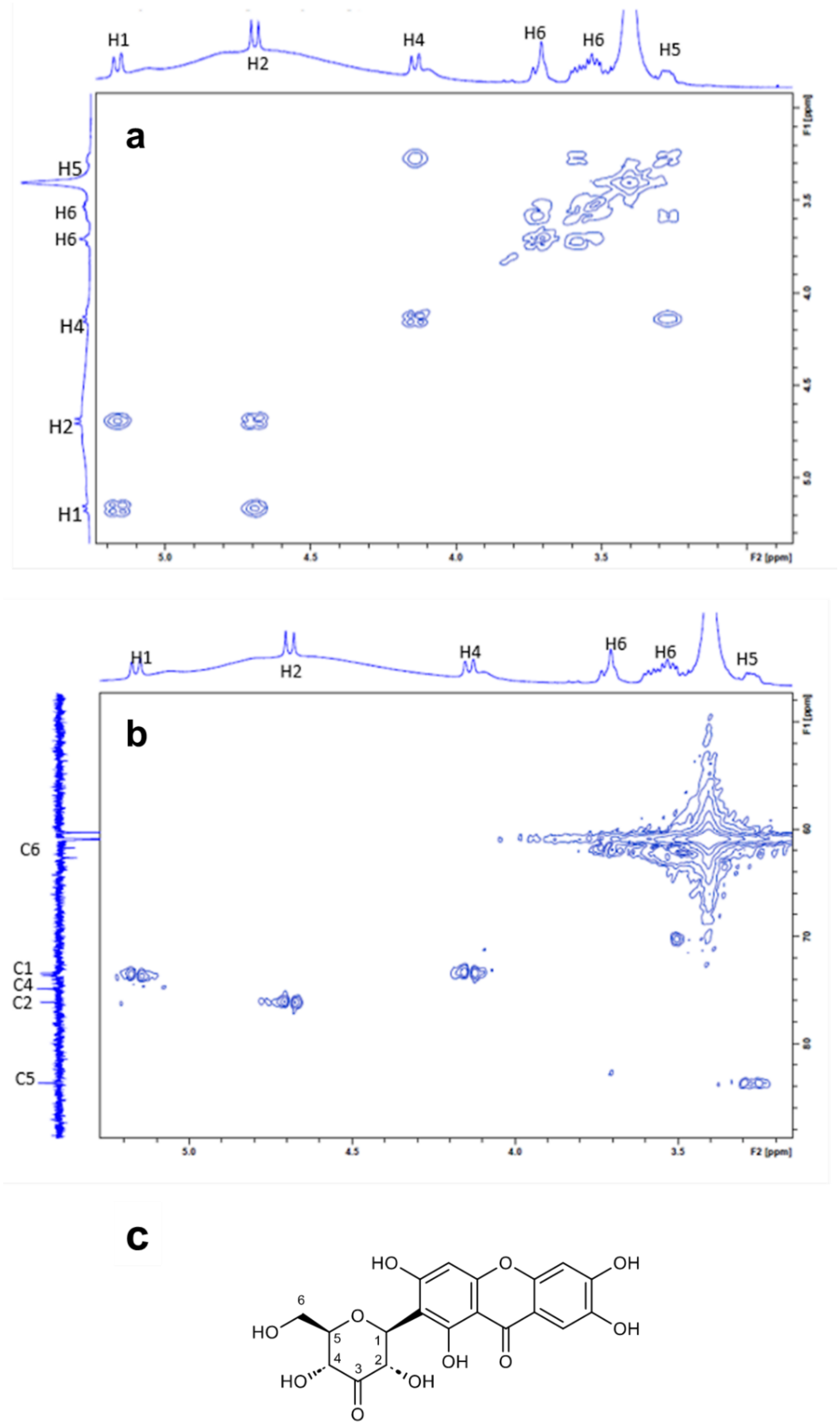

Supplementary Figure 7. NMR characterization of the oxidation product of Mang catalysed by *Ps*G3Ox. (a) 2D-COSY NMR spectrum. (b) HMQC NMR spectrum. (c) Chemical structure of 3-keto-Mang, the reaction product identified by NMR.

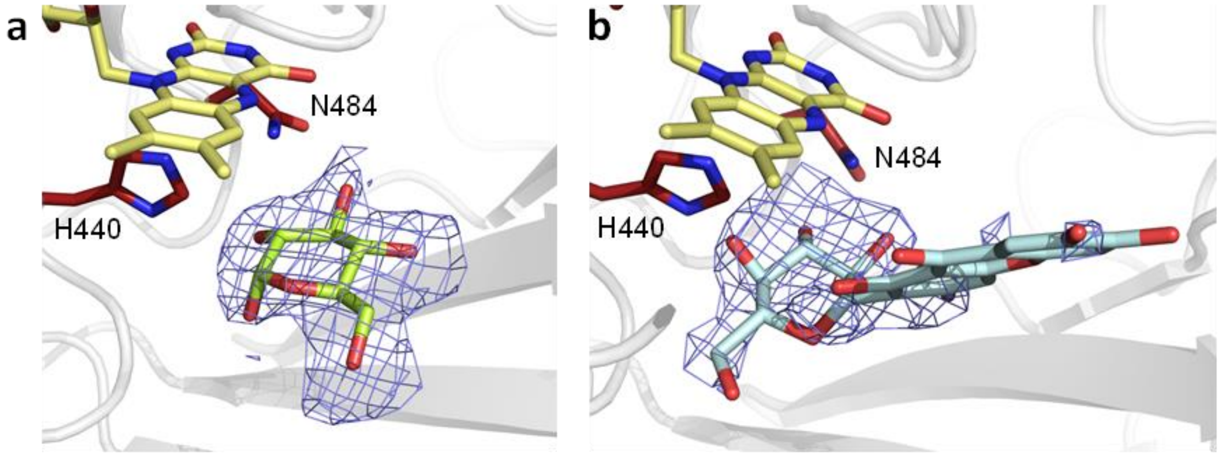

Supplementary Figure 8. Sigma-A weighted $2mF_o$-DF_c electron density maps of the substrates in *Ps*G3Ox-Glc and *Ps*G3Ox-Mang complexes crystal structures. Electron density contoured at 0.80 rmsd (blue) for (a) D-Glc and (b) Mang. The carbon atoms of the FAD, catalytic residues (H440 and N484), glucose and mangiferin are colored in yellow, dark red, green and light blue, respectively. The oxygen and nitrogen atoms are colored in red and blue, respectively.

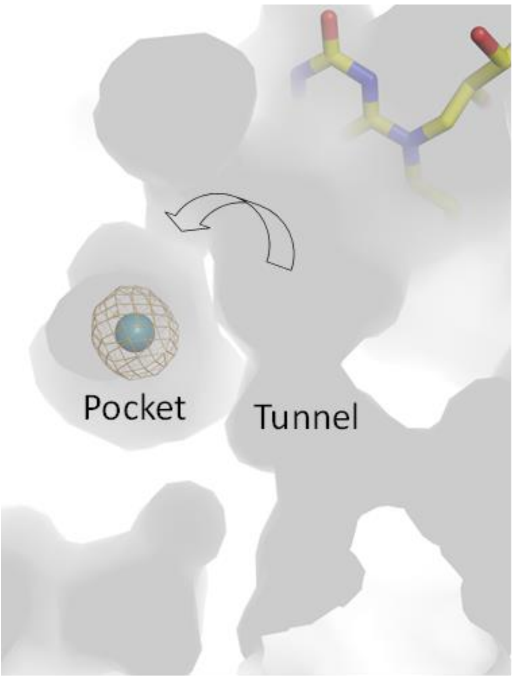

Supplementary Figure 9. Molecular pathways of *Ps*G3Ox that allow the access of the O_2 to the FAD^{N5}. Solvent-accessible surface, colored in gray, highlighting the hydrophobic tunnel and adjacent pocket. The arrow represents the possible diffusion path of the Kr from the tunnel to the pocket. The krypton atom is shown as a blue sphere and the electron density is contoured at 1.0 rmsd (orange). The carbon, oxygen and nitrogen atoms of the FAD, are colored in yellow, red and blue, respectively.

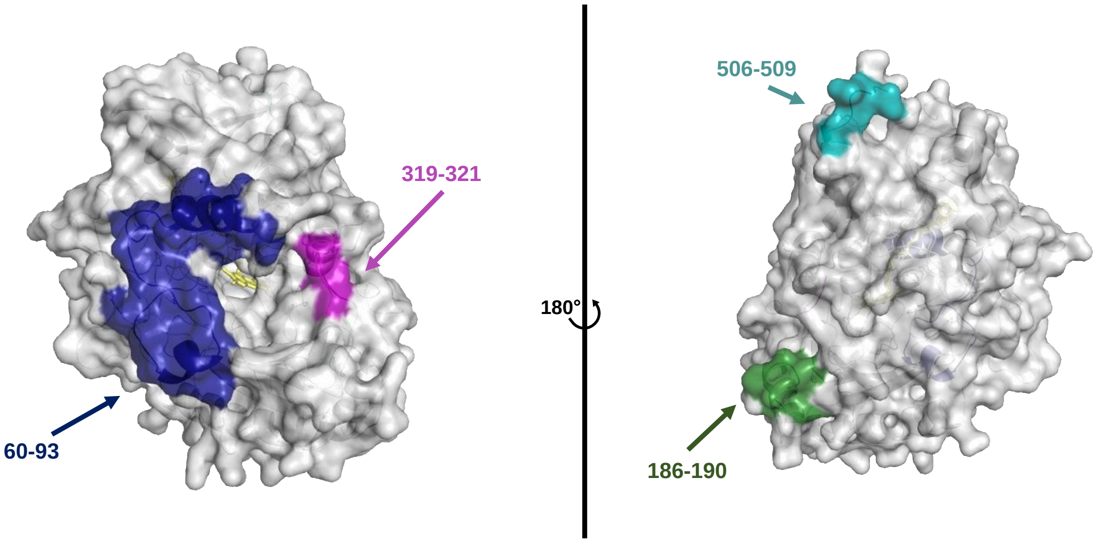

Supplementary Figure 10. Insertion regions in *Ps*G3Ox as compared to fungal P2Oxs. The *Ps*G3Ox monomeric form is shown as solvent-accessible surface colored gray. The insertion regions, 60-93, 186-190, 319-321, and 506-509, are colored in dark blue, green, magenta, and cyan, respectively. The FAD is shown as sticks colored in yellow.

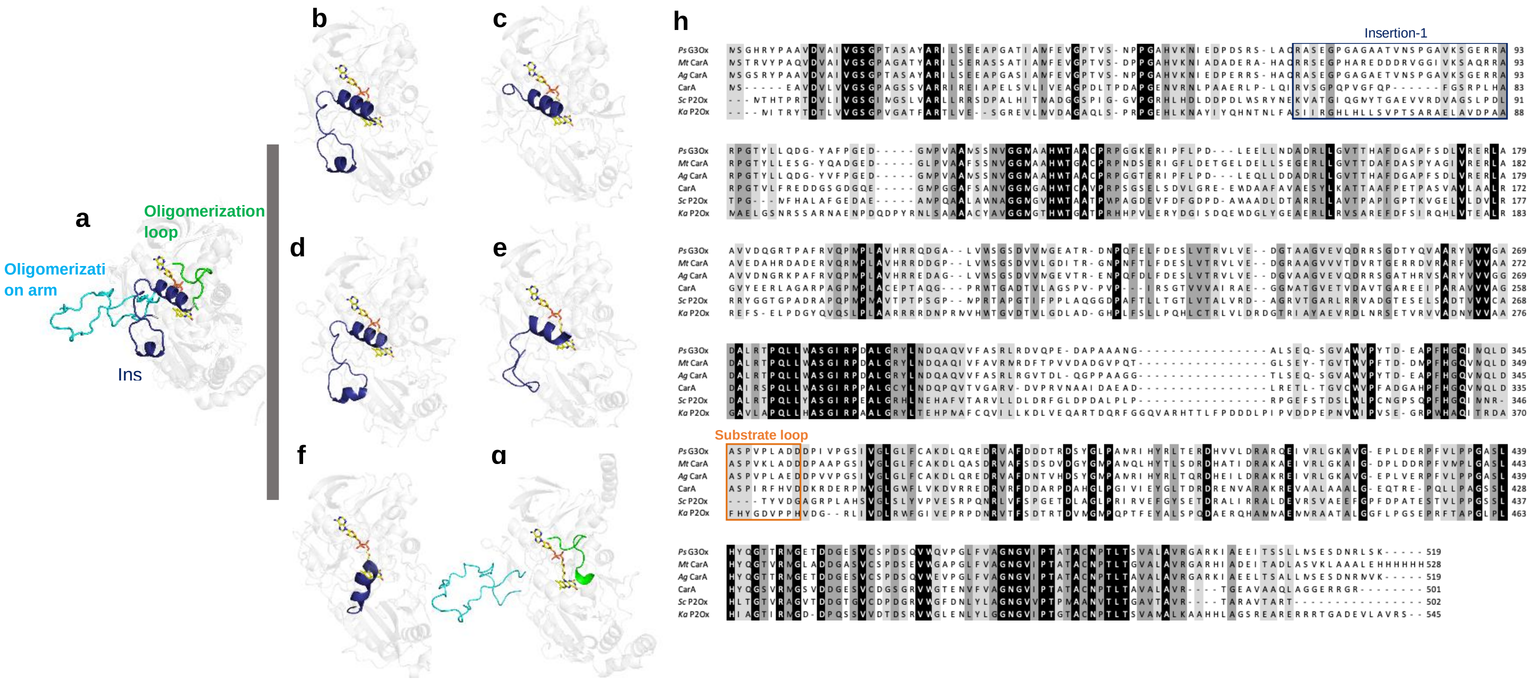

SUPPLEMENTARY FIGURE 11. Structural localization of the insertion-1 region in G3Ox and comparison to oligomerization features of P2Oxs. (a) Representation of the insertion-1 region (dark blue) in *Ps*G3Ox and oligomerization loop (green) and arm (cyan) in *Tm*P2Ox. Crystal structures of *Ps*G3Ox (b) and *Mt*CarA (c), and predicted alphafold model of (d) *Ag*CarA, (e) CarA, (f) *Sc*P2Ox, (g) *Ka*P2Ox. Enzyme segments equivalent to the *Ps*G3Ox insertion-1 are also present in the other bacterial enzymes with the exception of *Ka*P2Ox, which, similarly to the fungal ones, contains two oligomerization domains that could be relevant for its dimeric arrangement. The insertion-1 region is colored in dark blue in panels b-f. The oligomerization arm and loop are colored in cyan and green, respectively, in (g). In (h) Amino acid sequence alignment (primary sequence) of bacterial enzymes where the insertion-1 region and substrate loop are marked with blue and orange boxes; the amino acid composition is comparable in *Ps*G3Ox, *Ag*CarA, CarA, and *Mt*CarA. The amino acids that compose the substrate loop are less conserved among bacterial than fungal enzymes **(Figure 2h)**. The bacterial *Ka*P2Ox has a substrate loop with an amino acid composition ^{369}DAF<u>H</u>YG<u>D</u>V<u>P</u>^{377} comparable to the fungal *Tm*P2Ox ^{452}DAF<u>S</u>Y<u>G</u>AV<u>Q</u>^{460}.

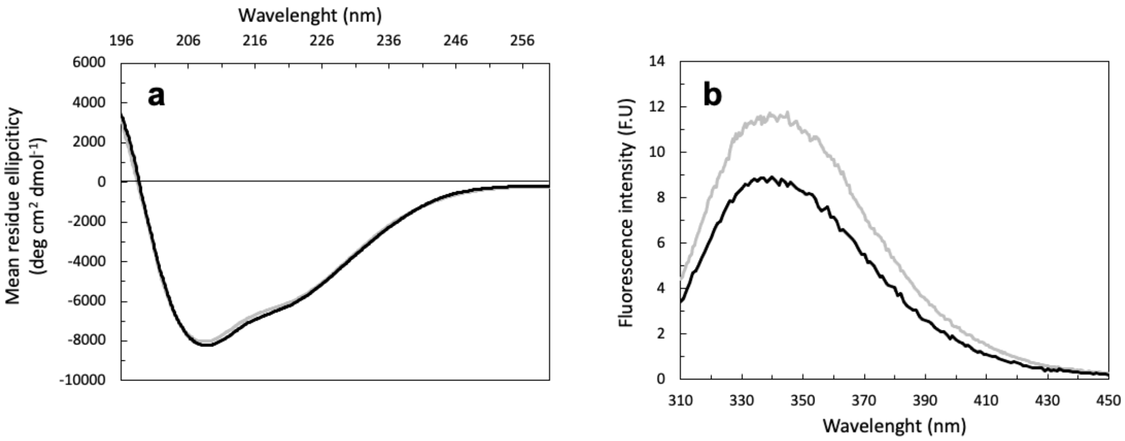

Supplementary Figure 12. The secondary and tertiary structure of *Ps*G3Ox after deflavination. **(a)** Circular dichroism spectra in the far-UV show the secondary structure of the partially FAD-loaded *Ps*G3Ox (black line) and the Apo *Ps*G3Ox (gray line). **(b)** Tryptophan fluorescence emission spectra of the partially FAD-loaded *Ps*G3Ox (black line) and the Apo preparation (gray line). Source data are provided as a Source Data file.

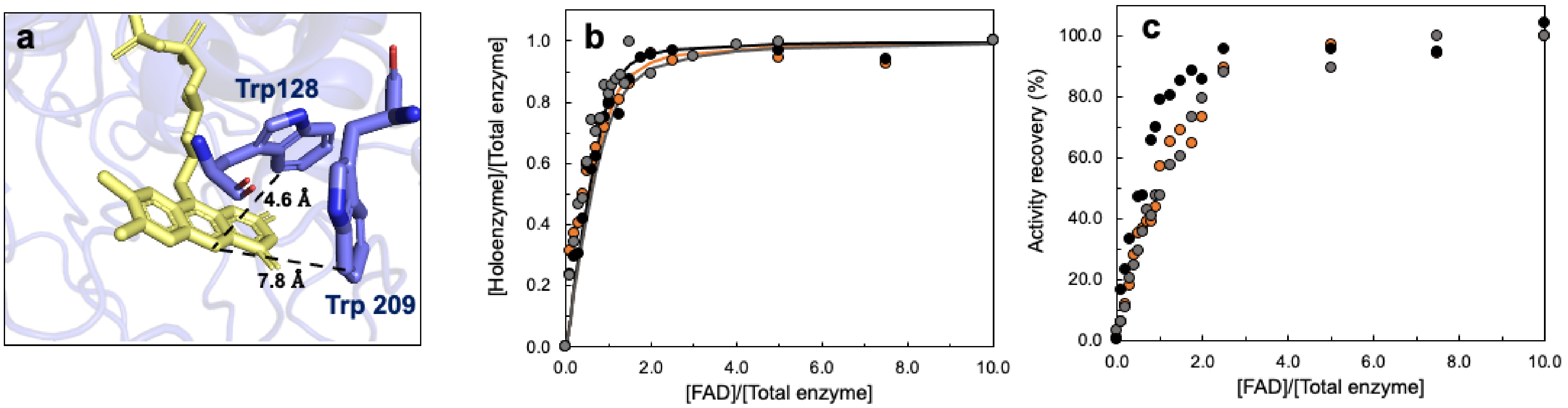

Supplementary Figure 13. ***In vitro*** **flavinylation of apo wild-type** ***Ps*****G3Ox. (a)** A highlight of the two tryptophan residues that could be quenched by the FAD attachment. Trp128 and Trp209 are located at 4.6 Å and 7.8, Å far from N5 of FAD, respectively. **(b)** Fraction of FAD-bond enzyme given by the normalized tryptophan fluorescence quenching as a function of the different ratios of FAD:Enzyme. The independent experiments (n=3) are represented in distinct colors. The solid line represents the theoretical fit of the experimental data to the one binding site model providing an estimated dissociation constant, $K_D = (2.0 \pm 0.7) \times 10^{-7}$ M and an affinity constant, $K_A = (5.3 \pm 1.9) \times 10^{6}$ M^{-1}. **(c)** Activity recovery experiments of apo-WT *Ps*G3Ox previously incubated with FAD in different ratios. The activity was measured using HRP-AAP/DCHBS coupled assay in 100 mM sodium phosphate buffer at pH 7.5, and the routine reaction mixture contained 1 M of D-Glc, 0.1 mM of AAP, 1 mM of DCHBS, and 8 U ml^{-1} of HRP. The independent experiments (n=3) are represented in distinct colors. Source data are provided as a Source Data file.

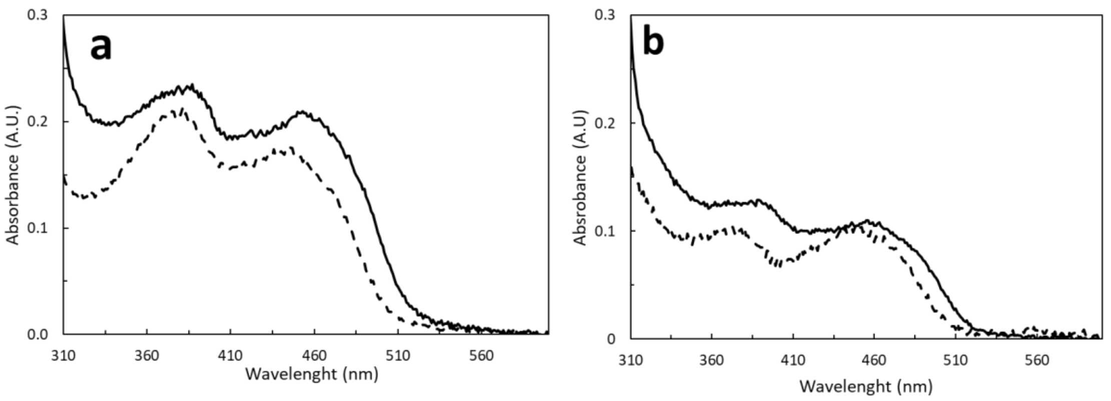

Supplementary Figure 14. FAD attachment analysis in different *Ps*G3Ox variants. UV-Vis spectra of purified enzyme preparations of (a) A125S and (b) A126T variants before (solid line) and after thermal denaturation (supernatant; dashed line). Source data are provided as a Source Data file.

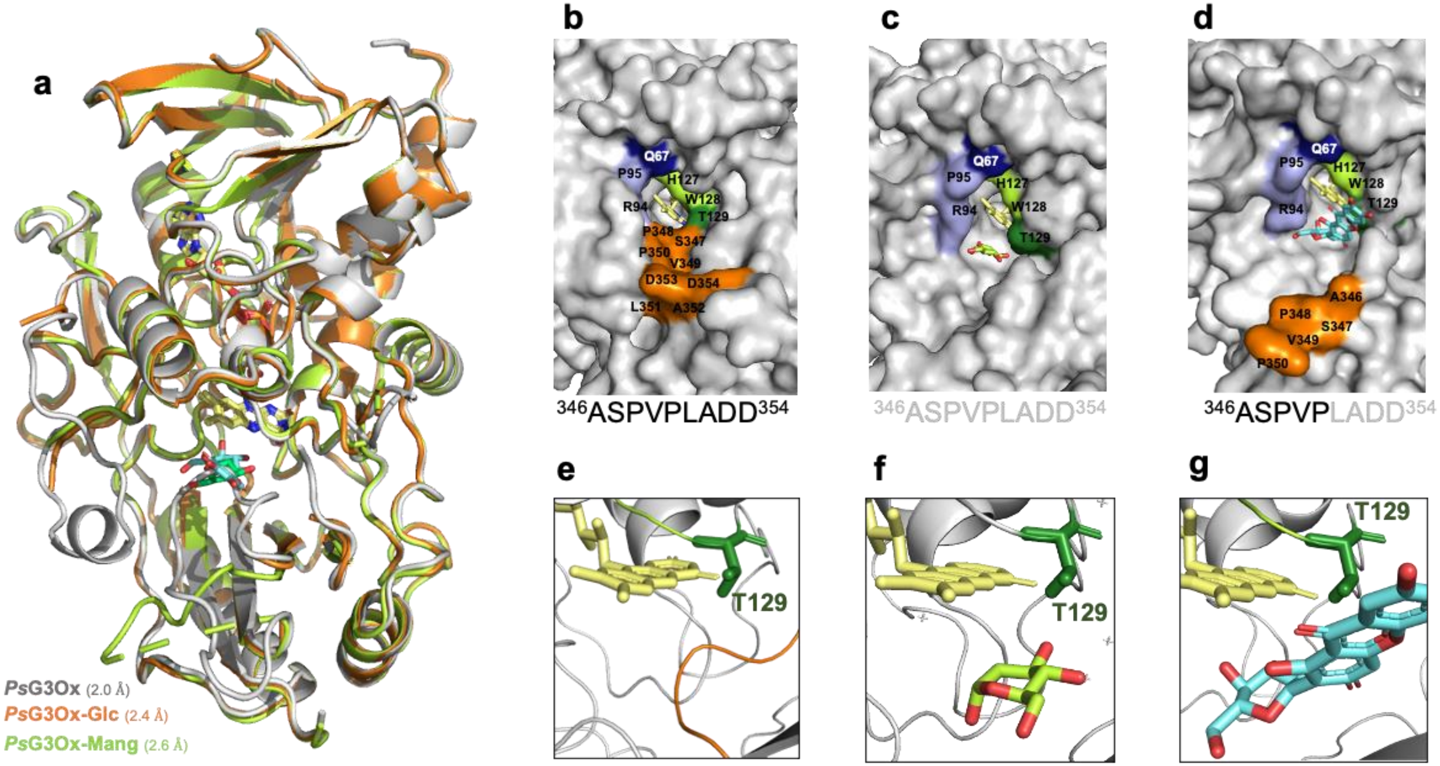

Supplementary Figure 15. **X-ray crystal structures of *Ps*G3Ox. (a)** Cartoon of the structural superposition of the crystal structures of substrate-free *Ps*G3Ox (grey) and structures in complex with D-Glc (orange) and Mang (light green). Solvent accessible surface representation of the active-site in (b) substrate-free *Ps*G3Ox, (c) *Ps*G3Ox-Glc and (d) *Ps*G3Ox-Mang complex crystal structures. The residues surrounding the FAD cavity are colored as in Figure 2d. The substrate loop amino acid composition is represented below the panels' b-d where the black letters represent the residues visible in the crystal structure and the gray ones the non-visible residues in the electron density maps. *Ps*G3Ox active site highlighting the conformation of the residue T129 in (e) substrate-free *Ps*G3Ox, (f) *Ps*G3Ox-Glc and (g) *Ps*G3Ox-Mang complexes. The FAD is shown as sticks in yellow color. The substrates D-Glc and Mang are shown as sticks coloured in green and cyan, respectively. In *Ps*G3Ox, the residue T129 establishes hydrogen bonds with FAD^{N5} (and $P348^{N}$) and also interacts with FAD^{N5} when the substrate loop is in a closed conformation similarly to the T169 in *Tm*P2Ox, (**Fig. S17a,d)**. However, contrary to the fungal enzymes in *Ps*G3Ox, the T129 appears to have the same configuration independently of the substrate loop conformation**.**

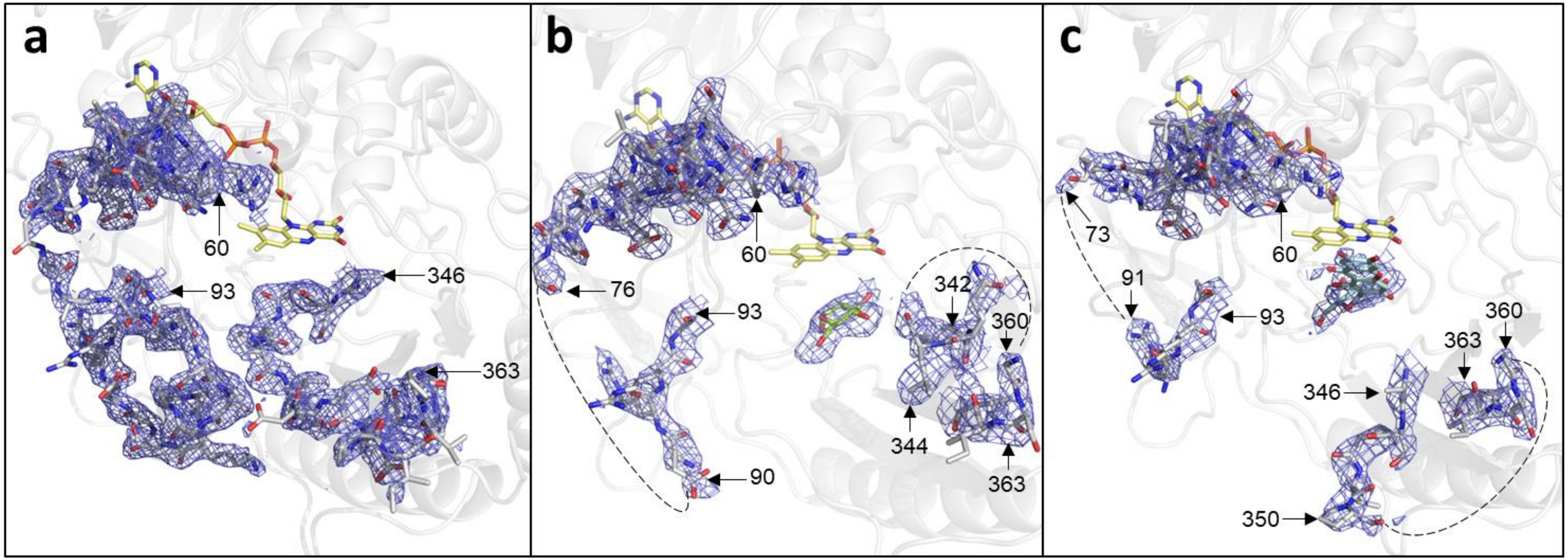

Supplementary Figure 16. Sigma-A weighted 2mF$_o$-DF$_c$ electron density maps of the insertion-1 and substrate loop in *Ps*G3Ox, *Ps*G3Ox-Glc and *Ps*G3Ox-Mang complexes crystal structures. Electron density contoured at 1.0 rmsd (blue) for the insertion-1 and substrate loop regions in (a) *Ps*G3Ox, (b) *Ps*G3Ox-Glc and (c) *Ps*G3Ox-Mang. The electron density is also shown for the substrates D-Glc and Mang. The carbon atoms of the FAD, D-Glc and Mang are colored in yellow, green and light blue, respectively. The oxygen and nitrogen atoms are colored in red and blue, respectively. The non-visible regions of the insertion-1, in *Ps*G3Ox-Glc (77-89) and in *Ps*G3Ox-Mang (74-90), and substrate loop in *Ps*G3Ox-Glc (345-359) and in *Ps*G3Ox-Mang (351-359), are represented as black dashed lines.

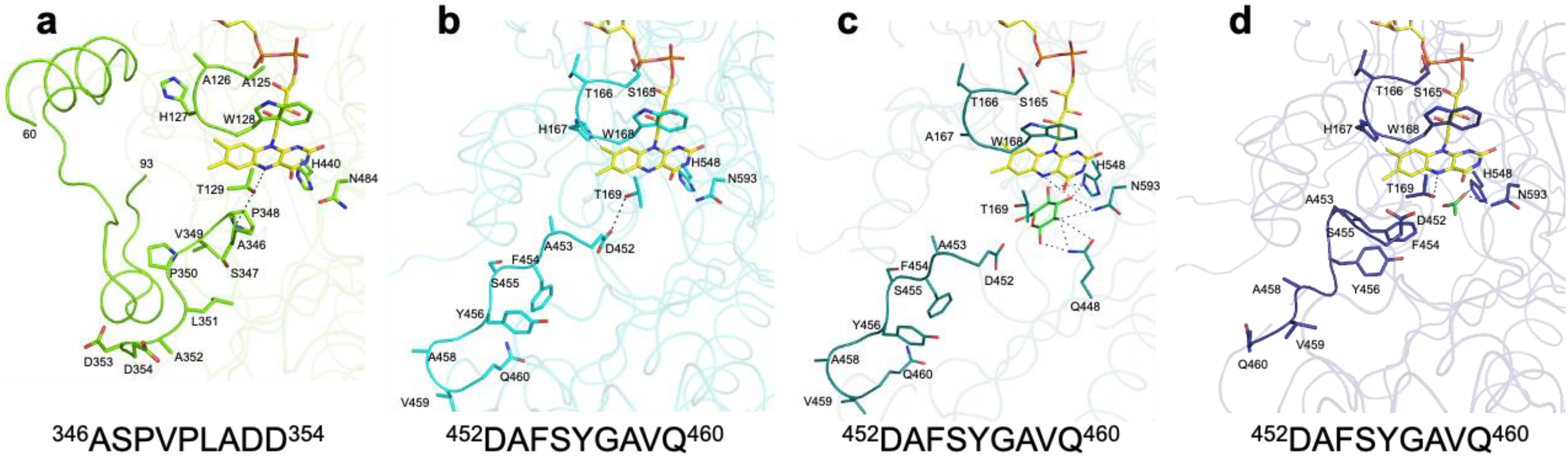

Supplementary Figure 17. Representing regions delimiting the FAD cavity in *Ps*G3Ox and fungal *T. multicolor* P2Ox. (a) Cartoon representation of the insertion-1, segment ^{125}AAHW^{128}, and substrate loop in closed conformation in substrate-free *Ps*G3Ox, in **(b)** *T. multicolor* P2Ox (substrate free; PDB 2IGK) in open conformation, in **(c)** *T. multicolor* P2Ox H167A in complex with 2-fluoro-2-deoxy-D-glucose (PDB 2IGO) that is oxidized in C3 position, shows an open conformation and in **(d)** *T. multicolor* P2Ox (with acetate, PDB 1TT0) in closed conformation of the substrate loop. The catalytic residues and residues part of the flavinylation motif and substrate loop are shown as sticks with carbon atoms colored in green, cyan, and dark blue for *Ps*G3Ox, 2IGK, and 2IGO, respectively. The FAD is shown as sticks with carbon, nitrogen, oxygen, and phosphorous atoms in yellow, blue, red and orange, respectively. The interatomic distances are shown as black dashed lines. The substrate loop amino acid composition for POxs is below the corresponding panels. The T169 residue, in *Tm*P2Ox, adopts different rotamer conformations and was suggested to trigger the transition between the different conformations of the substrate loop in fungal enzymes [2-4].

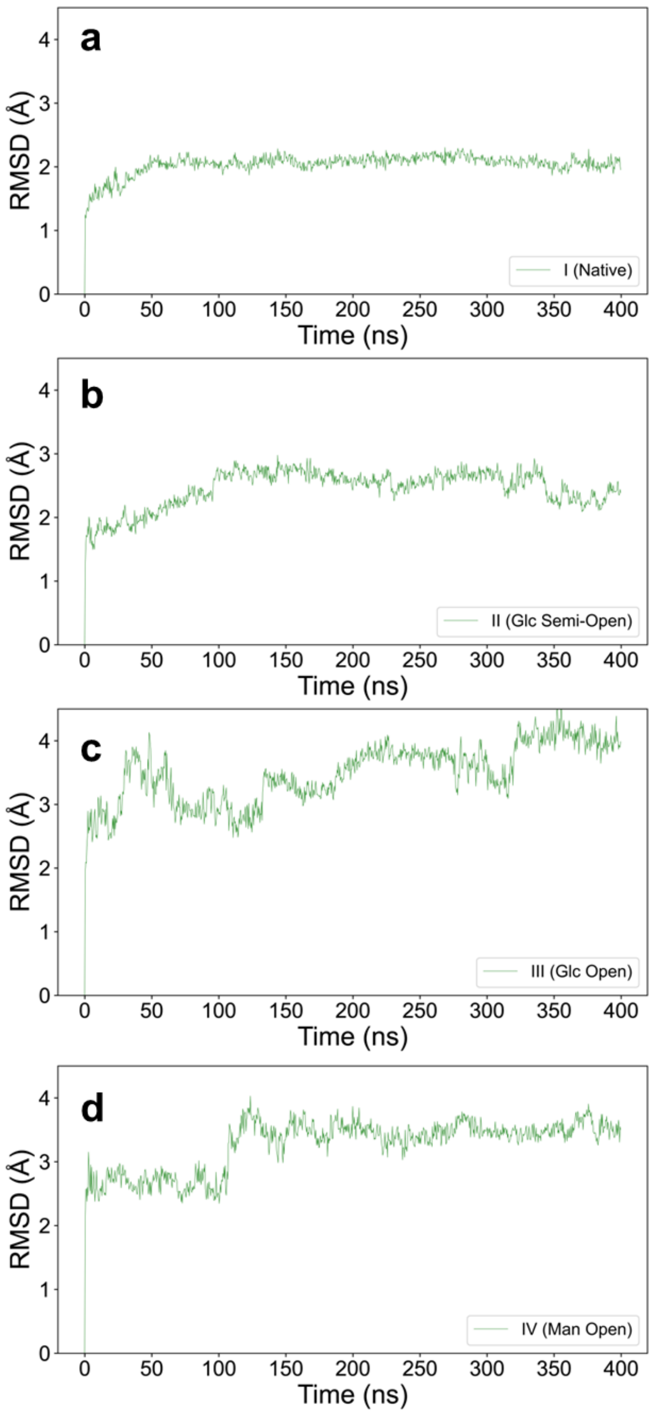

Supplementary Figure 18. RMSD (root-mean-square deviation, in Å) of Cα positions along the 400 ns conventional molecular dynamics simulations of Model I - IV (a-d) (see Material and Methods section for models' definition). The plots show convergence of the simulation for Model I, II and IV, whereas for Model III some fluctuations are seen as a result of glucose leaving the active site after 300 ns. Source data are provided as a Source Data file.

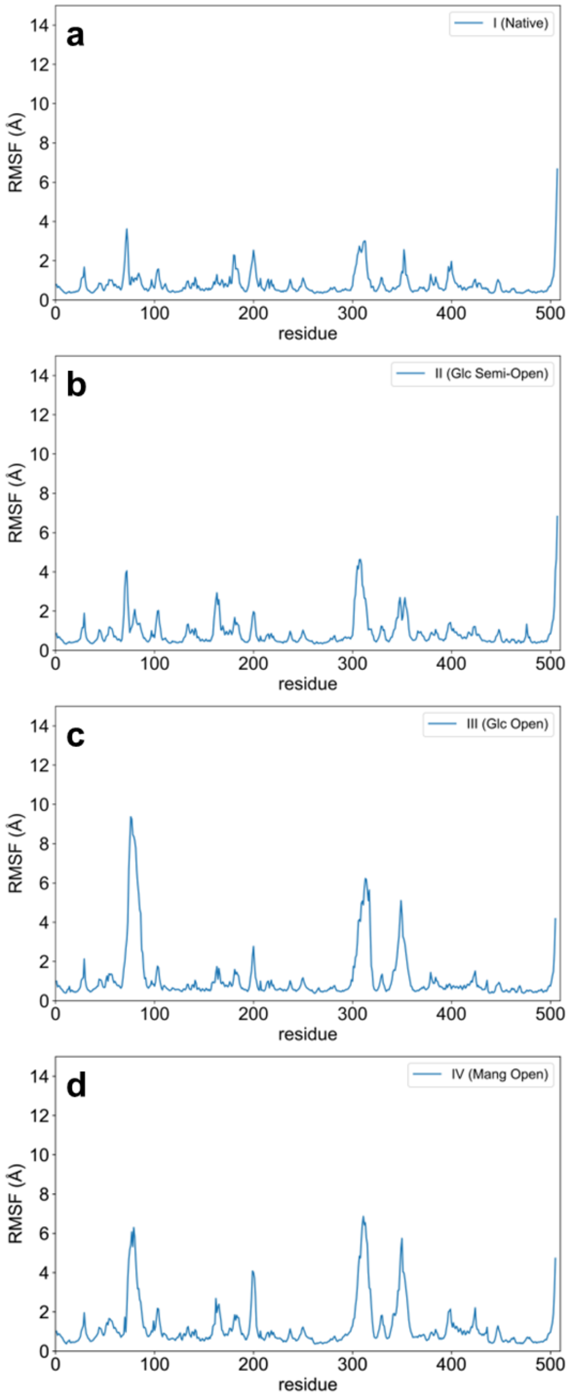

SUPPLEMENTARY FIGURE 19. RMSF (root-mean-square fluctuation, in Å) of Cα positions along the 400 ns conventional molecular dynamics simulations of Model I - IV (a-d) (see Material and Methods section for models' definition). The plots show higher fluctuation in the open state systems, mainly in the insertion-1 (60-93), in a solvent exposed loop at the enzyme's surface (~300-320) and in the substrate loop (346-354). Source data are provided as a Source Data file.

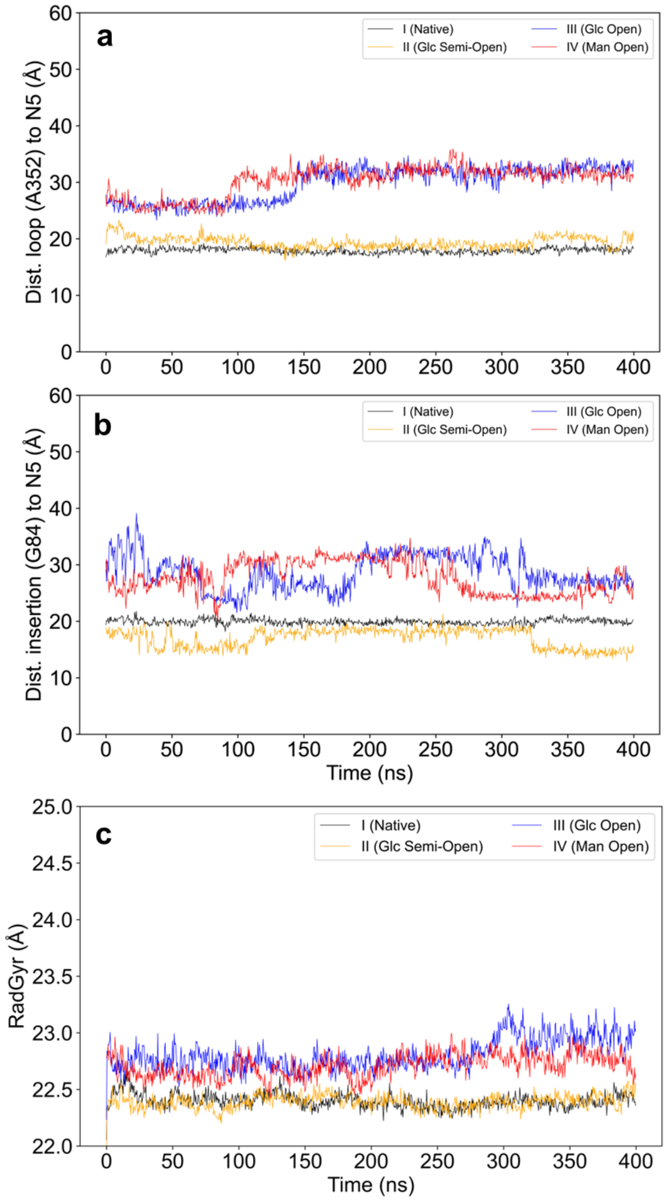

Supplementary Figure 20. Conformation of Substrate-loop (a), Inserion1-loop (b) and radius of gyration (c) along the 400 ns conventional molecular dynamics simulations: Model I (gray), II (orange), III (blue) and IV (red). The distance (in Å) between the N5 atom of FAD and residue A352 was monitored along the simulation as an indication of the conformation adopted by the substrate loop while the distance between G84 and N5 was measured to provide the information about insertion-1 loop conformation. In both graphics, shorter distances relate to closed conformations while long distances suggest open conformations. The insertion loop shows more changes along the simulations. For both loops, no transitions can be observed within these simulation times for closed and semi-open systems (the loops stay as in the corresponding initial conformation). Although for the simulations with initial open models, a further opening is observed, which could be related to the substrate displacement from the active site. As expected, a larger radius of gyration is observed for the open conformations. Source data are provided as a Source Data file.

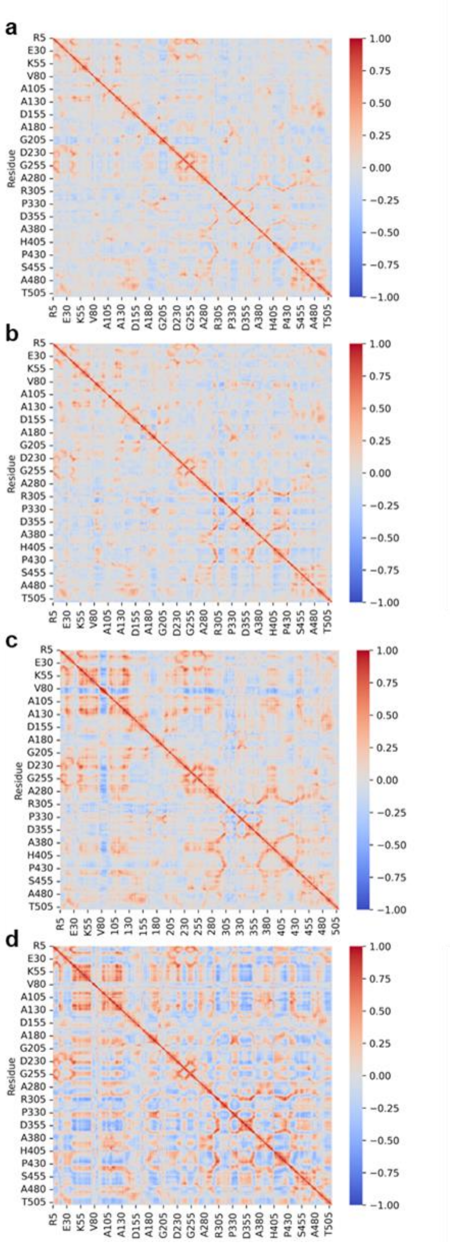

Supplementary Figure 21. Dynamical cross-correlation maps (DCCM) for the 400 ns conventional molecular dynamics simulations. (a) Model I (Native), (b) Model II (Glc Semi-open), (c) Model III (Glc Open) and (d) Model IV (Mang Open) (see Material and Methods section for models' definition). Source data are provided as a Source Data file.

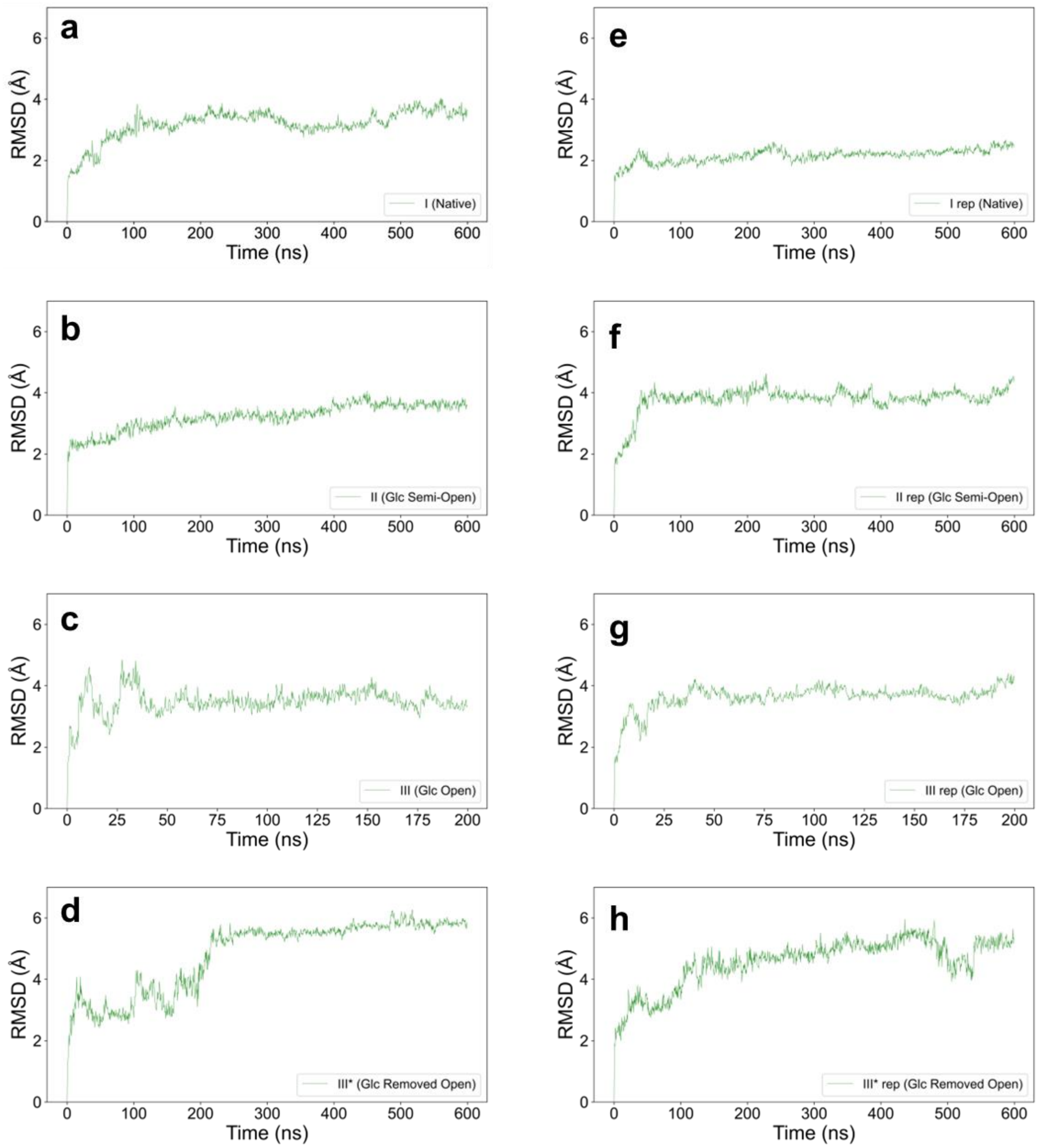

Supplementary Figure 22. RMSD of Cα positions along the GaMD simulations of Models I (a, e), II (b,f), III (c,g) and III* (d,h); see Material and Methods section for models' definition. For each model, two simulations with different distributions of the initial velocities were run to enhance the conformational space sampled. For Model III, the simulations were stopped at 200 ns since D-Glc left the binding site (see Supplementary Figure 26). Despite being GaMDs, the graphs show relatively stable RMSD as the trajectories advance. The sudden increase in the first model III* replicate, which starts from open conformations of the loops and with D-Glc removed, corresponds to the closing of the substrate loop. Source data are provided as a Source Data file.

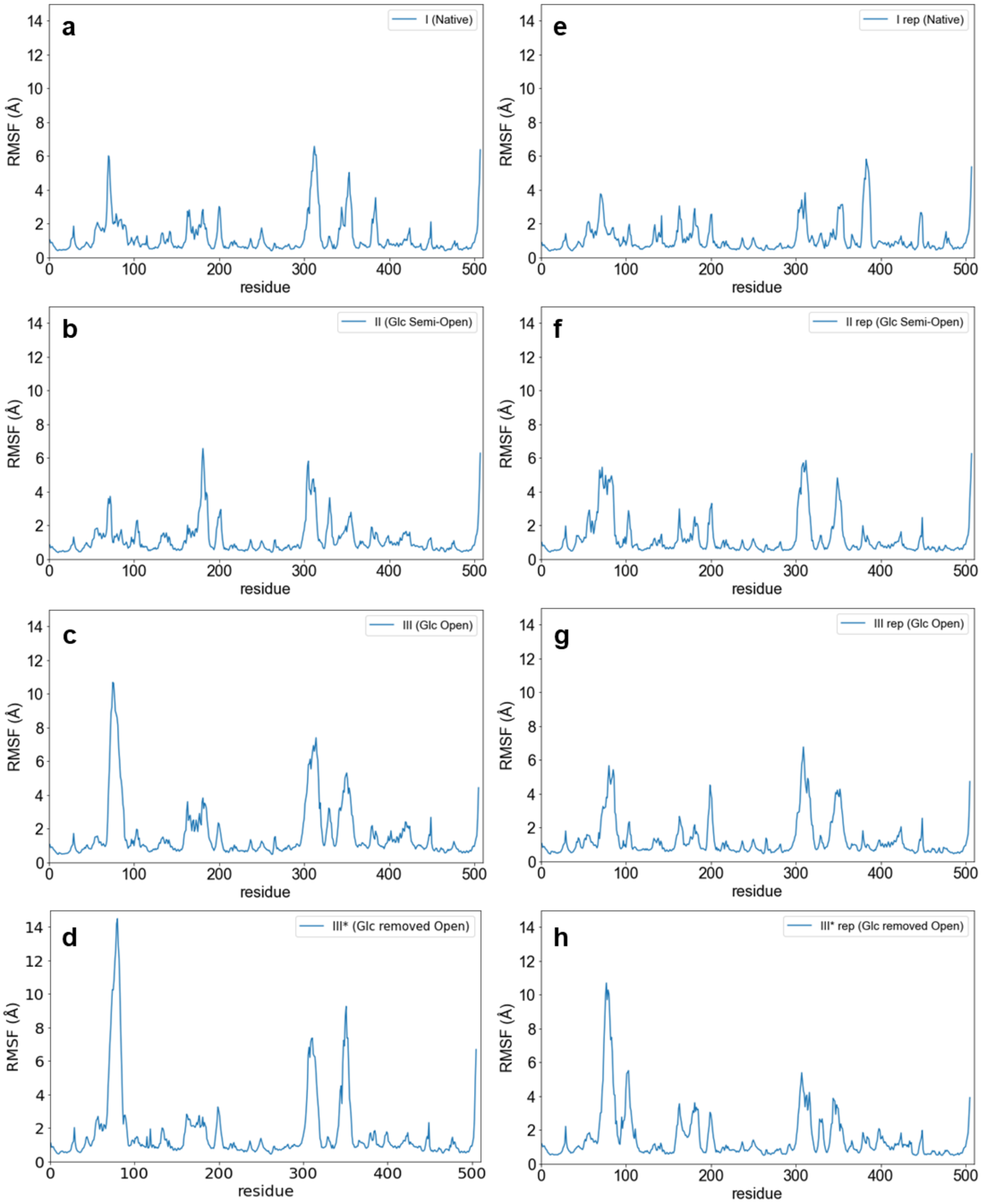

SUPPLEMENTARY FIGURE 23. RMSF of Cα positions along the 600 ns GaMD simulations of Model I (a,e), II (b,f), III (c,g) and III* (d,h) (see material and methods section for models' definition). For each model, two simulations with different distributions of the initial velocities were run to enhance the conformational space sampled. For Model III, the simulations were stopped at 200 ns since D-Glc left the binding site. The plots show higher fluctuation in the open state systems, mainly in the insertion-1 (60-93), in a solvent exposed loop at the enzyme's surface (~300-320) and in the substrate loop (346-354). Source data are provided as a Source Data file.

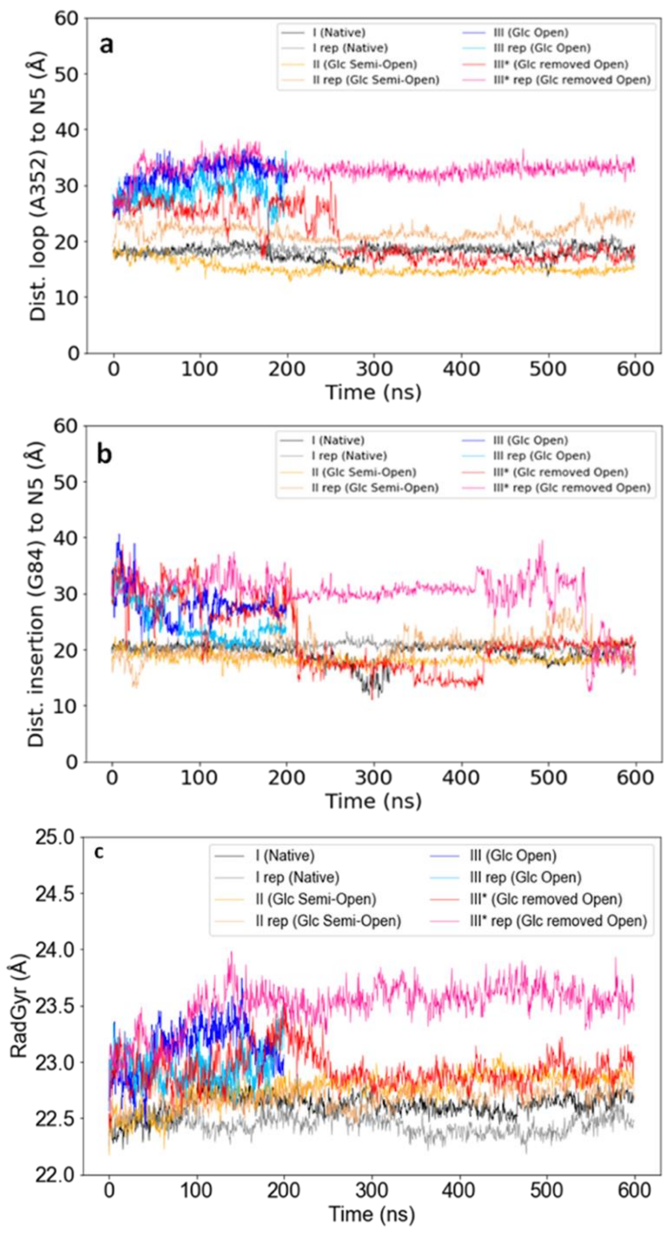

Supplementary Figure 24. Conformation of Substrate-loop (a), Inserion1-loop (b), and radius of gyration (c) along the GaMD simulations. The two simulations for each model are represented: for Model I (black and gray), II (orange and brown), III (blue and light blue) and III* (red and pink), see materials and methods section for models' definition. The distance (in Å) between the N5 atom of FAD and residue A352 was monitored along the simulation as an indication of the conformation adopted by the substrate loop while the distance between G84 and N5 was measured to provide the information about insertion-1 loop conformation. In both graphics, shorter distances relate to closed conformations while long distances suggest open conformations. For both loops, no transitions are observed within these simulation times for a closed system (the loops stay as in the corresponding initial conformation). Although for both loops in the semi-open and open system, more conformational changes are observed. For Model III the simulations were stopped at 200 ns because D-Glc left the binding site (see Supplementary Figure 26). Source data are provided as a Source Data file.

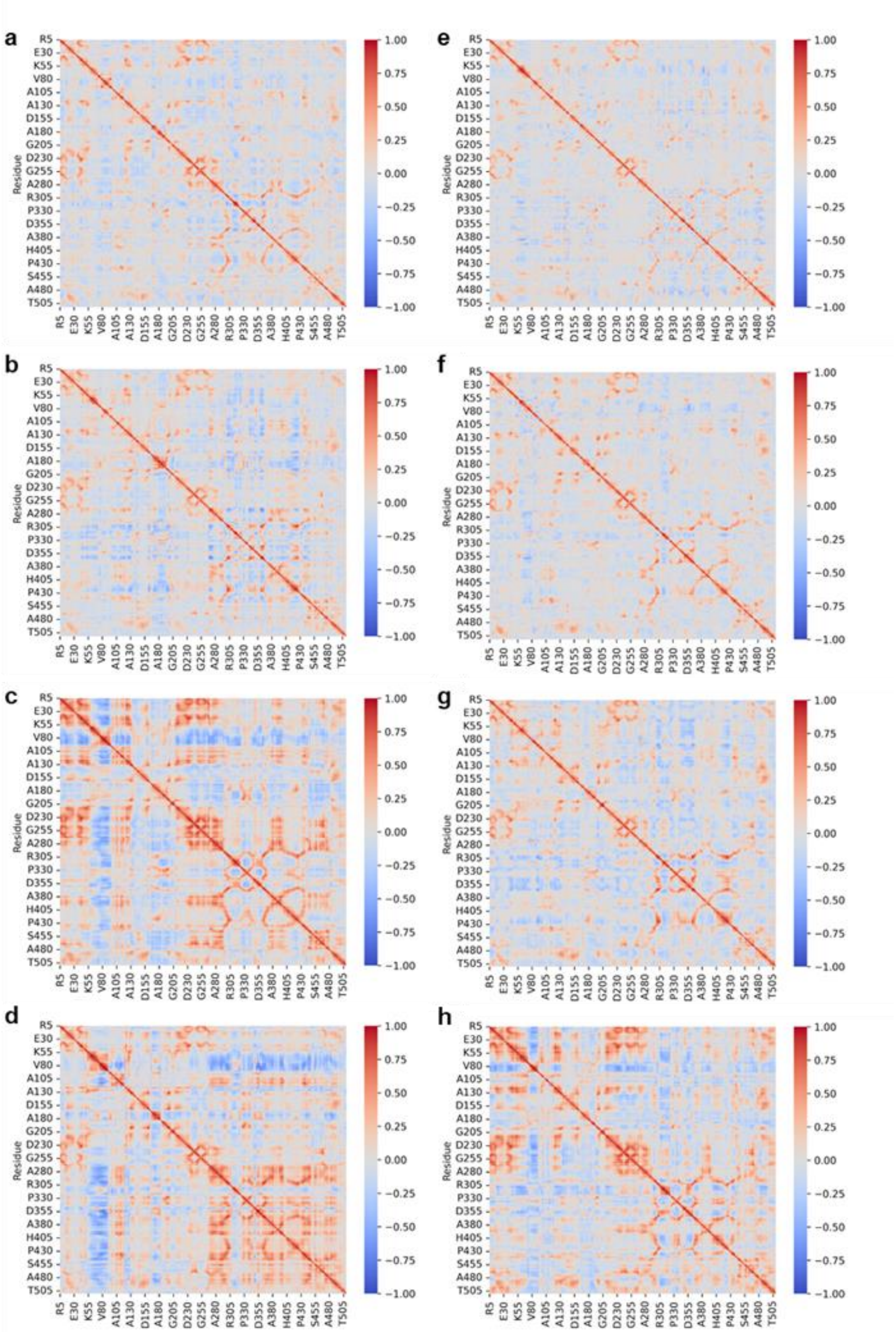

Supplementary Figure 25. Dynamical cross-correlation maps (DCCM) for the GaMD simulations of Model I (Native) (a, e), II (Glc Semi-Open) (b, f), III (Glc Open) (c, g) and III* (Glc removed Open) (d,h) (see material and methods section for further models' definition). For each model, two simulations with different distributions of the initial velocities were run to enhance the conformational space sampled. Source data are provided as a Source Data file.

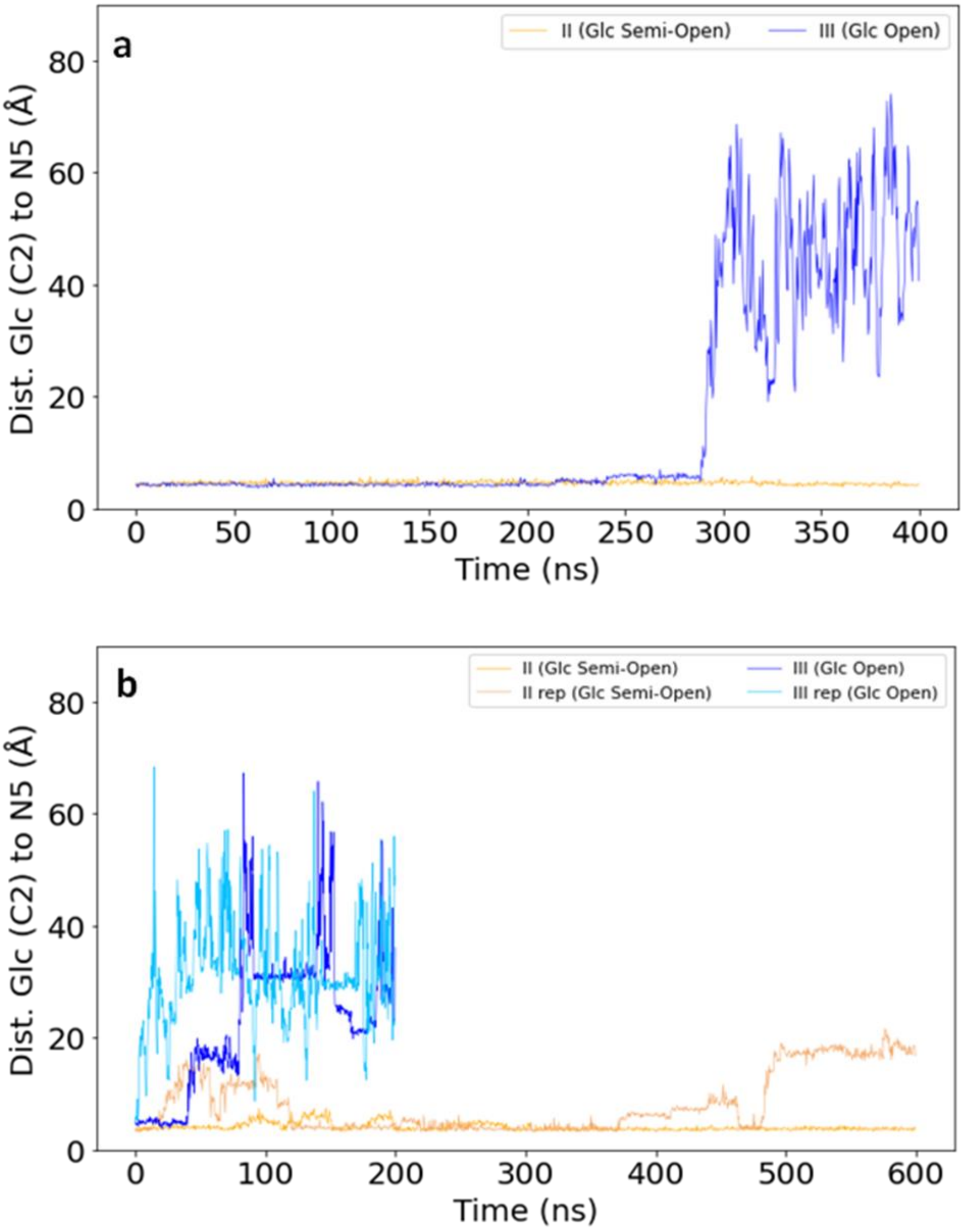

Supplementary Figure 26. Distance between the C2 of D-Glc and N5 of FAD during the cMDs (a) and GaMD (b) simulations for the model II and model III systems (see materials and methods section for models' definition). In (b) the two simulations that were run for each model are represented (for model II in yellow and light orange; for Model III in blue and light blue). Source data are provided as a Source Data file.

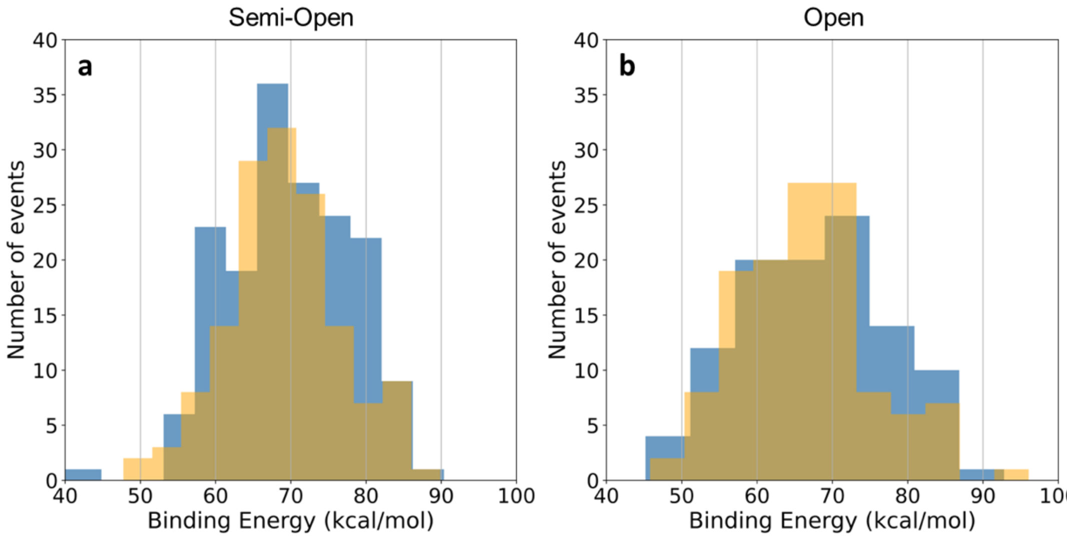

Supplementary Figure 27. Local dockings of D-glucose to wild-type *Ps*G3Ox with a semi-open (a) and open (b) substrate loop conformations. Full histograms of D-glucose's binding energies (in kcal/mol). Docking results were filtered as C2 and C3 binding modes or discarded, according to their catalytic distances to FAD's N5 and H440. Data in blue and yellow represent docking results in a binding mode suitable for C2 and C3 oxidation, respectively. A higher Yasara's binding energies correspond to better docking results [5]. Source data are provided as a Source Data file.

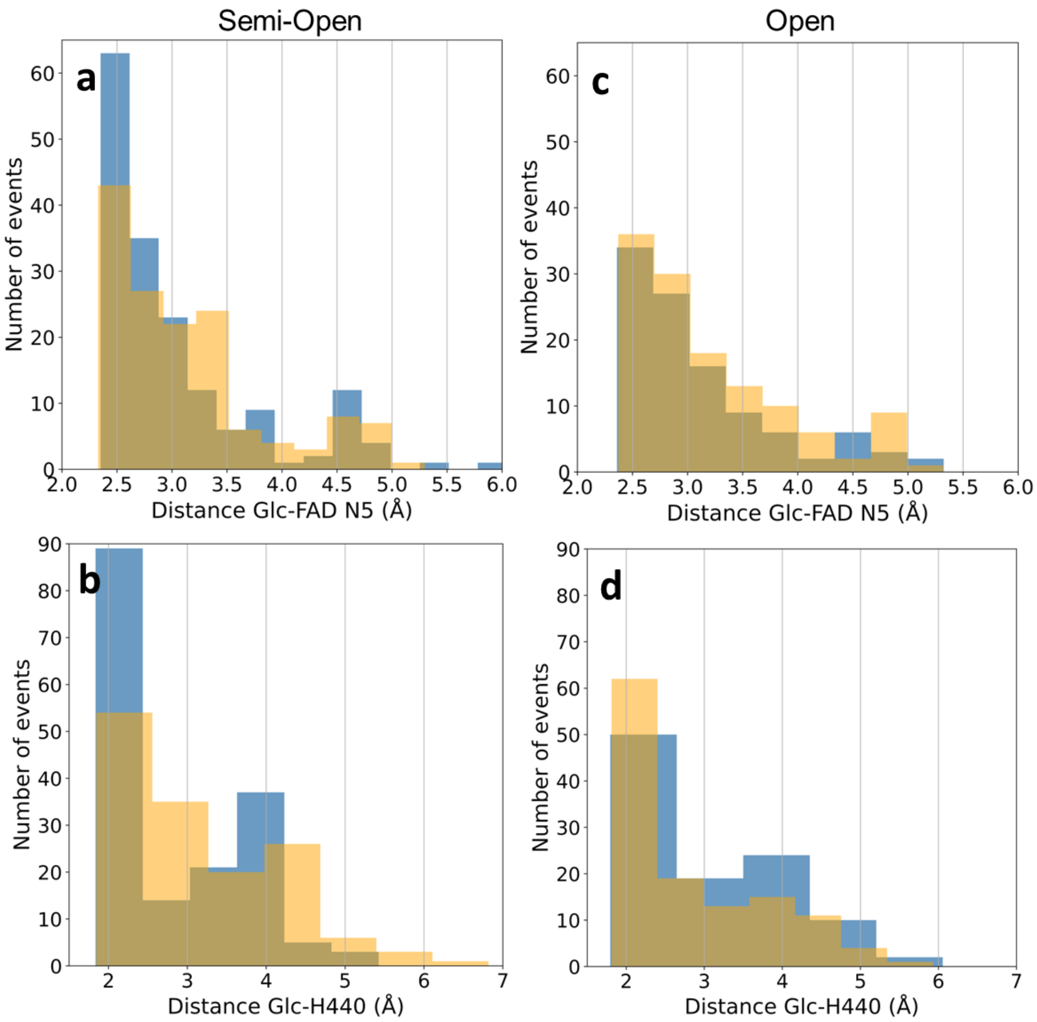

Supplementary Figure 28. Local dockings of D-glucose to wild-type *Ps*G3Ox with a semi-open (a-b) and open (c-d) substrate loop conformations. Full histograms of the distances (in Å) between D-Glc and the FAD(N5) or H440. Docking results were filtered as C2 and C3 binding modes or discarded, according to their catalytic distances to FAD's N5 and H440. Data in blue and yellow represent docking results in a binding mode suitable for C2 and C3 oxidation, respectively. Source data are provided as a Source Data file.

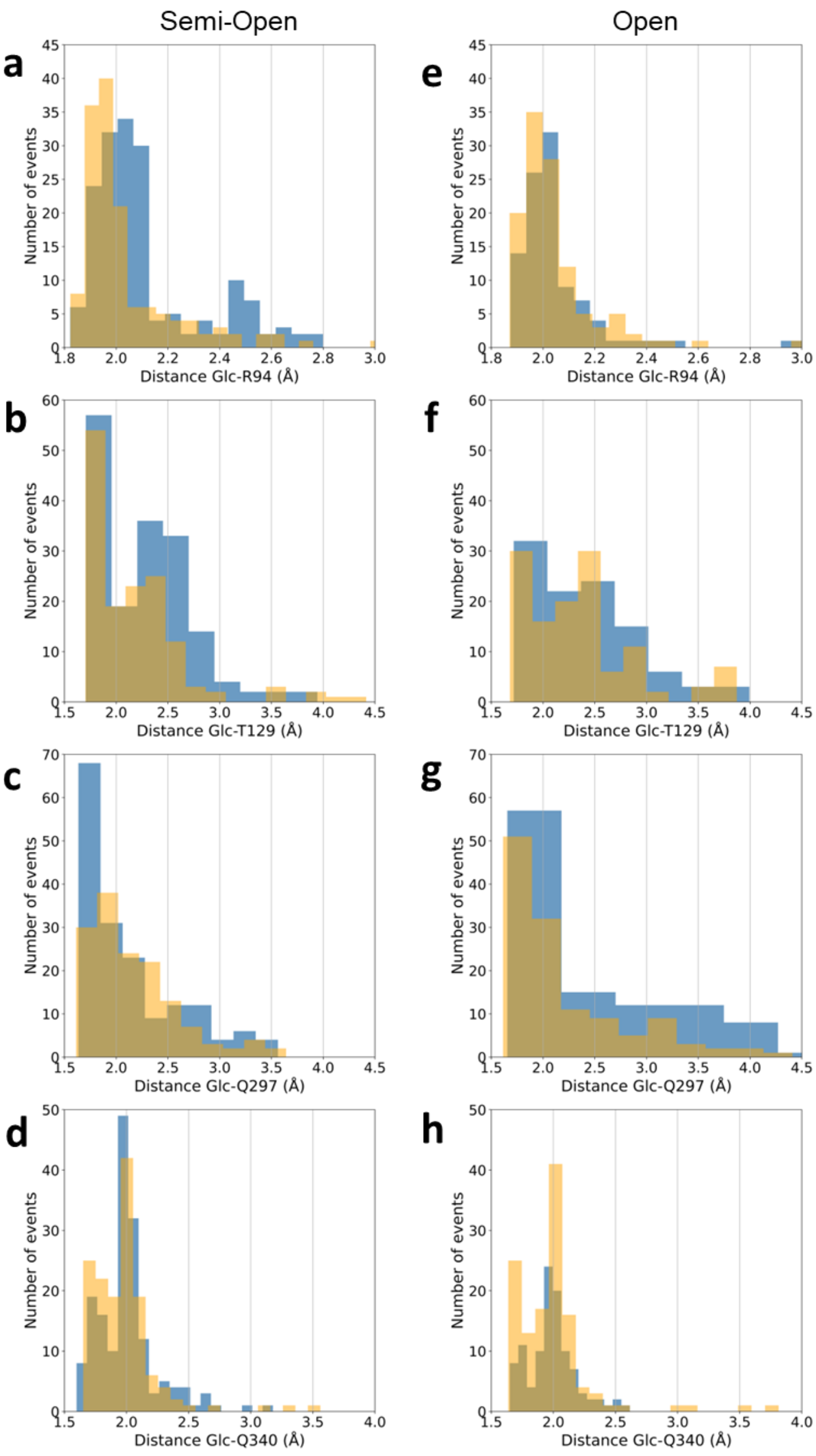

Supplementary Figure 29. Local dockings of D-Glc to wild-type *Ps*G3Ox with semi-open (a-d) and open (e-h) substrate loop conformations. Full histograms of the distance (in Å) between D-glucose and non-catalytic interacting residues. Docking results were filtered as C2 and C3 binding modes or discarded, according to their catalytic distances to FAD's N5 and H440. Data in blue and yellow represent docking results in a binding mode suitable for C2 and C3 oxidation, respectively. Source data are provided as a Source Data file.

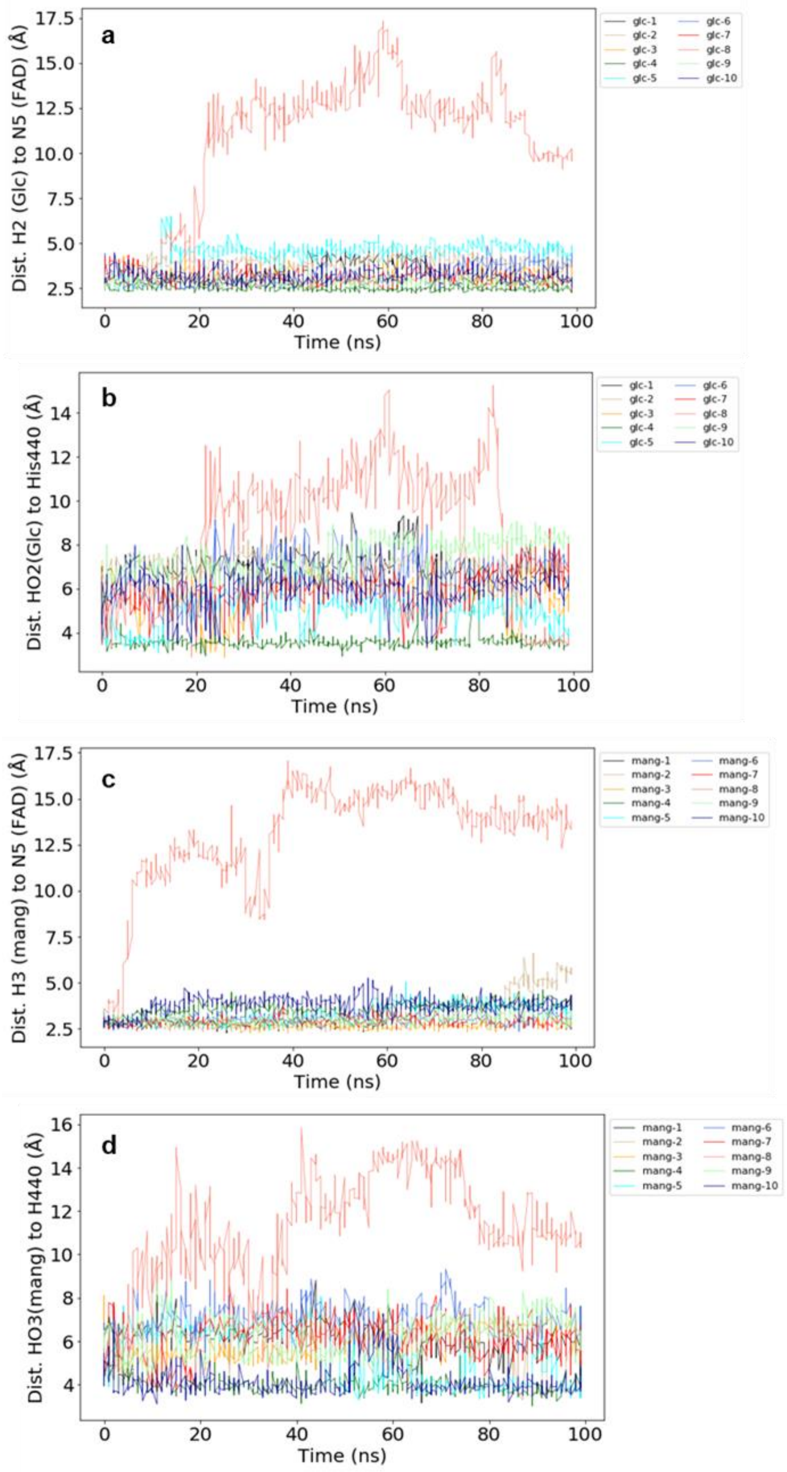

Supplementary Figure 30. Molecular dynamics simulations (10 trajectories of 100 ns length each) of the enzyme:substrate complex for D-Glc (a,b) and Mang (c,d) used in the MMPBSA free energy calculations. Catalytic distances D-Glc^{H2}–FAD^{N5}, D-Glc^{HO2}–His440^{N}, Mang^{H3}–FAD^{N5} and Mang^{HO3}–His440^{N} are monitored along the unrestricted simulations. Except for few simulations in which the ligand unbinds, both substrates remain in the active site with H2/H3 to FAD^{N5} distances (for D-Glc and Mang, respectively) consistent with catalysis. Notice that the distance to the catalytic base H440 of the proton to be abstracted is longer, which supports a mechanism initiated by hydride transfer. Source data are provided as a Source Data file.

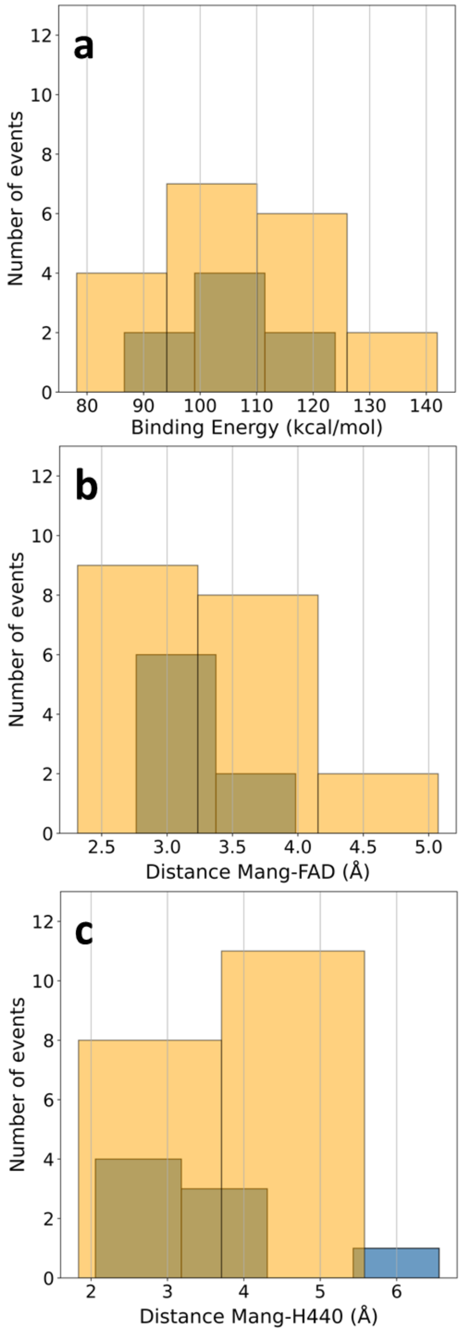

Supplementary Figure 31. Local dockings of Mang to wild-type *Ps*G3Ox with an open substrate loop conformation. Full histograms of Mang binding energy (in kcal/mol) **(a)** and distance (in Å) between Mang and FAD(N5) **(b)** or H440 **(c)**. Docking results were filtered as C2 and C3 binding modes or discarded, according to their catalytic distances to FAD's N5 and H440. Data in blue and yellow represent docking results in a binding mode suitable for C2 and C3 oxidation, respectively. Higher Yasara's binding energies correspond to better docking results[5]. Source data are provided as a Source Data file.

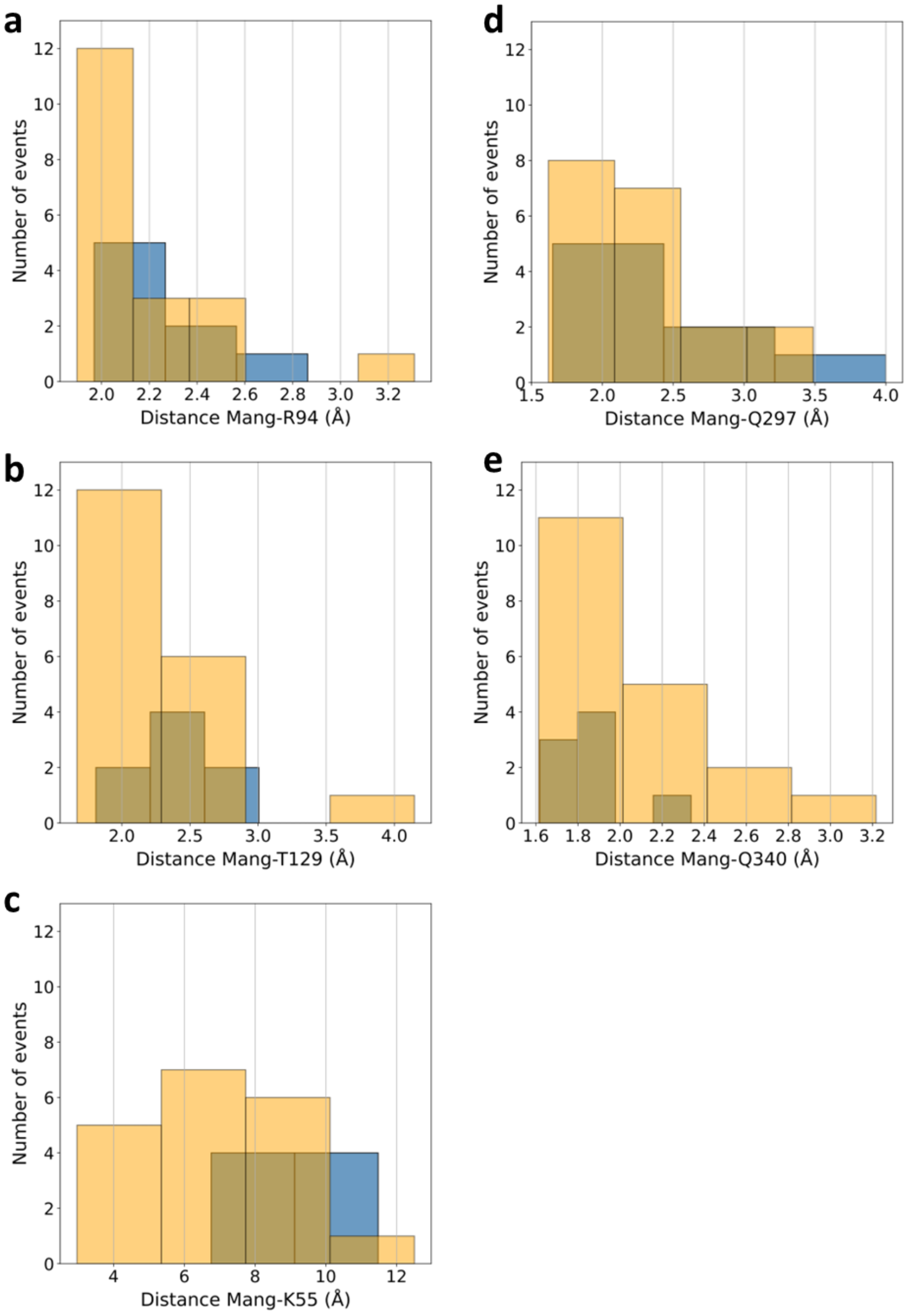

Supplementary Figure 32. Local dockings of Mang to wild-type *Ps*G3Ox with an open substrate loop conformation. Full histograms of the distances (in Å) between Mang and the non-catalytic interacting residues (**a-e**). Docking results were filtered as C2 and C3 binding modes or discarded according to their catalytic distances to FAD's N5 and H440. Data in blue and yellow represent docking results in a binding mode suitable for C2 and C3 oxidation, respectively. Source data are provided as a Source Data file.

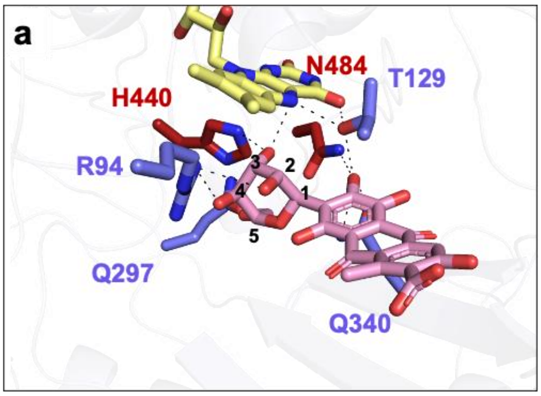

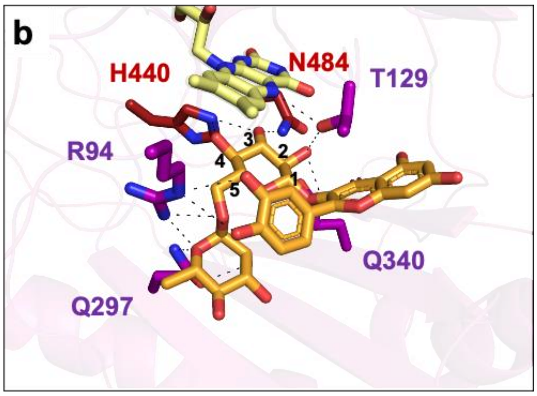

Supplementary Figure 33. Docking models highlighting the interactions of carminic acid (a), and rutin (b) with *Ps*G3Ox. The FAD is shown as sticks colored in yellow. The catalytic residues (H440 and N484) are shown as sticks colored in dark red. All other interacting residues are shown as sticks in purple or magenta for carminic acid and rutin dockings, respectively. The carminic acid and rutin molecules are shown as sticks colored in pink and orange, respectively. The hydrogen bonds are shown as black dashed lines. Carminic acid binds in a non-catalytically competent manner: H2/C2 is relatively well oriented towards FAD^{N5}, but it is the OH3 that interacts with catalytic H440 (HO3/O3-NE: 1.82/2.78 Å; O3-HO3-NE: 160.9º), and no interaction with the catalytic residue N484 was observed. In the case of rutin, the dockings predict binding in an orientation compatible with catalysis at C3 (H3/C3-N5:2.30/3.32 Å; C3-H3-N5: 155.7º; HO3/O3-NE: 1.82/2.78 Å; HO4-NE: 2.58 Å).

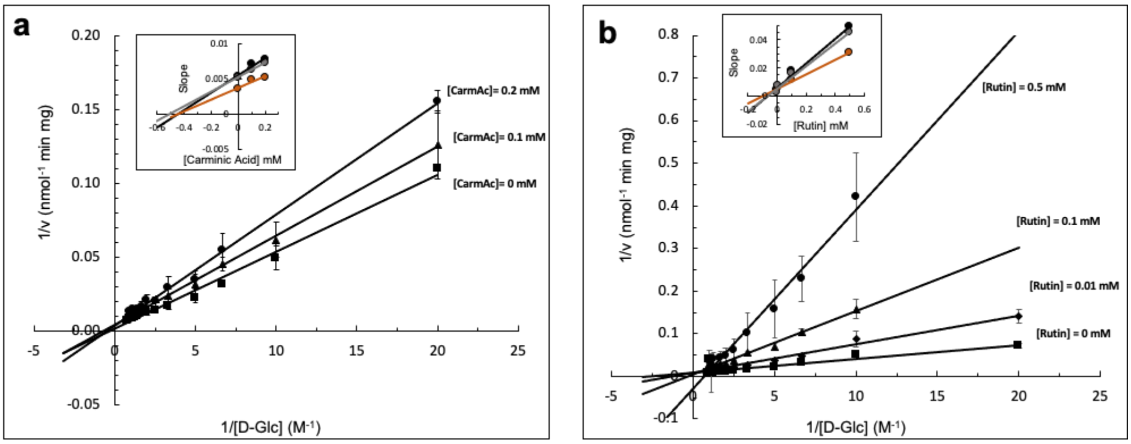

Supplementary Figure 34. Lineweaver-burk plots of *Ps*G3Ox inhibition by (a) carminic acid and (b) rutin. The activity was measured using HRP-AAP/DCHBS coupled assay in 100 mM sodium phosphate buffer at pH 7.5, 37 ºC and the routine reaction mixture contained 0-1 M of D-Glc, 0.1 mM of AAP, 1 mM of DCHBS, 8 U ml^{-1} of HRP and 0-0.2 mM carminic acid or 0-0.5 mM rutin. The independent experiments (n=3) are represented as mean ± SD. The inset are the secondary plots of the slopes obtained in the Lineweaver Burk plots against inhibitor concentration where the independent experiments (n=3) are represented in distinct colors. The estimated inhibition constant, K_i, were 0.10 ± 0.05 mM for rutin and 0.45 ± 0.02 mM for carminic acid. Source data are provided as a Source Data file.

2. Supplementary Tables

Supplementary Table 1. Optimization of the colorimetric assay to measure the oxidase activity of *Ps*G3Ox. The rate of consumption by *Ps*G3Ox of $ABTS^{\bullet+}$ and N-(4-antipyryl)-3-chloro-5-sulfonate-p-benzoquinone-monoimine, a pink chromogen, was determined in the presence and absence of molecular oxygen using D-Glc as the electron donor. Source data are provided as a Source Data file.

	Rate of consumption (nmol min^{-1} mg^{-1})	
	$ABTS^{\bullet+}$	Pink chromogen
+ O_2	368 ± 46	25 ± 1
- O_2	441 ± 74	77 ± 12

Supplementary Table 2. Apparent steady-state catalytic parameters for D-Glc and O_2 for POxs in the literature.

Origin	Microorganism	Enzyme	D-Glc[a]			O_2[b]			References
			k_{cat} (s^{-1})	K_m (M)	k_{cat}/K_m ($M^{-1}s^{-1}$)	k_{cat} (s^{-1})	K_m (M)	k_{cat}/K_m ($M^{-1}s^{-1}$)	
Bacterial	*Pseudarthrobacter siccitolerans*	***Ps*G3Ox**	0.19	0.46	0.45	0.14	5.8×10^{-6}	19.9×10^{3}	This work
	Pantoea ananatis Sd-1	***Pa*P2Ox**	ND	-	-	ND	-	-	6
	Kitasatospora aureofaciens	***Ka*P2Ox**	15.4	1.5×10^{-3}	1.0×10^{4}	32.4	1.1×10^{-3}	3.0×10^{4}	7
	Microbacterium sp. 5-2b	**CarA**	nd	-	-	ND	-	-	8
	Arthrobacter globiformis	***Ag*CarA**	nd	-	-	ND	-	-	8
	Microbacterium trichothecenolyticum	***Mt*CarA**	nd	-	-	ND	-	-	8
	Streptomyces canus	***Sc*P2Ox**	> 0.38	> 2.0	> 0.19	0.02	6.0×10^{-5}	3.3×10^{2}	9
Fungal	*Phlebiopsis gigantea*	***Phg*P2Ox**	40.5	1.2×10^{-3}	3.4×10^{4}	ND	-	-	10
	Trametes multicolor	***Tm*P2Ox**	54.0	0.7×10^{-3}	7.3×10^{4}	71.0	9.0×10^{-5}	7.9×10^{5}	11
	Tricholoma matsutake	***TmaP2Ox***	111.0	1.3×10^{-3}	8.7×10^{4}	ND	-	-	12
	Peniophora gigantea	***Peg*P2O**	56	1.1×10^{-3}	5.0×10^{4}	ND	-	-	13
	Peniophora sp	***Ps*P2Ox**	9.4	5.0×10^{-3}	1.8×10^{3}	ND	-	-	14
	Phanerochaete chrysosporium	***Pc*P2Ox**	83.1	0.8×10^{-3}	9.9×10^{4}	109.0	1.2×10^{-3}	8.9×10^{4}	15
	Lyophyllum shimeji	***Ls*P2Ox**	6.9	0.3×10^{-3}	2.2×10^{4}	ND	-	-	16
	Aspergillus nidulans	***An*P2Ox**	35.4	1.8×10^{-3}	2.0×10^{4}	ND	-	-	17
	Aspergillus oryzae	***Ao*P2Ox**	1.5	2.9×10^{-3}	0.5×10^{3}	ND	-	-	17
	Irpex lacteus	***Il*P2Ox**	33.8	0.7×10^{-3}	4.6×10^{4}	ND	-	-	18

[a] Molecular oxygen was used as an electron acceptor; [b] D-Glc was used as an electron donor (except for *Sc*P2Ox where xylose was used); ND-not determined; nd-not detected

Supplementary Table 3. Identification of Mang oxidation product by NMR. Summary of ^{1}H (400 MHz) and ^{13}C (100.61 MHz) chemical shift in DMSO-d6 for Mang sugar moiety and for its oxidation product catalyzed by *Ps*G3Ox.

	**Position**	**^{1}H**, ***δ*H** (ppm)	**Multiplicity**, J(MHz)	**^{13}C, *δ*C** (ppm)
**Mangiferin**	1	4.60	d, 10.01	73.7
	2	4.06	t, 9.30	70.7
	3	[3.25-3.09]	m	79.6
	4	[3.25-3.09]	m	70.8
	5	[3.25-3.09]	m	82.2
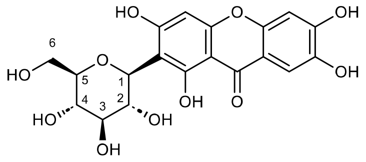	6	3.69	m	61.9
	6	3.38	m	61.9
**Oxidation product**	1	5.17	d, 10.06	73.4
	2	4.69	d, 10.05	76.1
	3	nd	-	209
	4	4.14	d, 9.82	73.6
	5	3.27	m	83.8
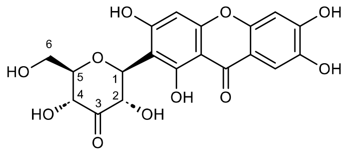	6	3.71	m	61.8
	6	3.55	m	61.8

nd- Not detected.

Supplementary Table 4. X-ray data collection and refinement statistics. Values in parentheses belong to the highest resolution shell.

	***Ps*G3Ox**	***Ps*G3Ox-Glc**	***Ps*G3Ox-Mang**
**Data collection**			
Beamline	ESRF ID23-2	ESRF ID30A-1	ALBA XALOC
Wavelength (Å)	0.8731	0.9654	0.97926
Space group	*C* 2 2 2*₁*	*C* 2 2 2*₁*	*C* 2 2 2*₁*
Unit cell parameters (Å)	*a* = 78.5, *b* = 78.8, *c* = 151.3	*a* = 78.3, *b* = 78.5, *c* = 150.2	*a* = 76.7, *b* = 78.9, *c* = 147.3
Resolution (Å)	39.39 – 2.01 (2.11 – 2.01)	75.11 – 2.35 (2.46 – 2.35)	73.67 – 2.60 (2.70 – 2.60)
Nr. observations	361063 (51690)	94766 (9314)	87843 (13386)
Nr. unique reflections	31572 (5031)	18894 (2511)	14110 (2229)
Completeness (%)	99.8 (99.6)	96.2 (90.8)	99.5 (97.9)
Multiplicity	11.4 (10.3)	5.0 (3.7)	6.2 (6.0)
Mosaicity (°)	0.43	0.43	0.48
$CC_{1/2}$ (%)[a]	99.6 (65.3)	97.3 (42.4)	98.5 (49.5)
R_{sym} (%)[b]	15.2 (181.3)	18.8 (185.9)	17.8 (44.9)
R_{meas} (%)[c]	17.9 (188.6)	28.4 (154.9)	20.8 (63.0)
$R_{p.i.m.}$ (%)[d]	4.6 (26.4)	14.7 (47.4)	7.8 (20.2)
<I/σ(I)>	9.57 (0.99)	4.11 (0.94)	6.99 (1.16)
Wilson B ($Å^2$)	42.0	50.9	44.0
V_M ($Å^3$ Da^{-1})	1.83	1.80	2.01
Estimated solvent content (%)	32.7	31.8	39.0
**Refinement**			
R_{work} (%)[e]	19.9	24.5	22.8
R_{free} (%)[e]	24.4	26.2	28.2
rmsd for bond lengths (Å)	0.003	0.002	0.002
rmsd for bond angles (°)	0.542	0.465	0.467
Structure <*a.d.p.*> ($Å^2$)	52.2	64.9	30.8
Ramachandran plot			
Residues in favored regions (%)	98.0	93.5	96.4
Residues in allowed regions (%)	2.0	6.5	3.6
Residues in disallowed regions (%)	0	0	0
PDB code	7QF8	7QFD	7QVA

[a] $CC_{1/2}$ = Percentage of correlation between intensities from random half-datasets.

[b] $R_{sym} = \frac{\sum_{hkl}\sum_i |I_i(hkl) - \langle I(hkl)\rangle|}{\sum_{hkl}\sum_i I_i(hkl)}$, where Ii(hkl) is the observed intensity and <I(hkl)> is the average intensity of multiple observations from symmetry-related reflection.

[c] $R_{meas} = \frac{\sum_{hkl}\sqrt{\frac{N(hkl)}{N(hkl)-1}}\sum_{i=1}^{N}|I_i(hkl) - \langle I(hkl)\rangle|}{\sum_{hkl}\sum_i I_i(hkl)}$, where N(hkl) is the data multiplicity, Ii(hkl) is the observed intensity and <I(hkl)> is the average intensity of multiple observations from symmetry-related reflections. It is an indicator of the agreement between symmetry related observation.

[d] $R_{p.i.m.} = \frac{\sum_{hkl}\sqrt{\frac{1}{N(hkl)-1}}\sum_{i=1}^{N}|I_i(hkl) - \langle I(hkl)\rangle|}{\sum_{hkl}\sum_i I_i(hkl)}$, where N(hkl) is the data multiplicity, Ii(hkl) is the observed intensity and <I(hkl)> is the average intensity of multiple observations from symmetry-related reflections. It indicates the precision of the final merged and averaged data set.

[e] R_{work} refers to the actual working data set used in refinement, while R_{free} refers to a cross-validation set that is not directly used in refinement and is therefore free from refinement bias.

Supplementary Table 5. Amino acid residues that limit the tunnel and cavity in the substrate-free *Ps*G3Ox, *Ps*G3Ox-glucose (*Ps*G3Ox-Glc), and *Ps*G3Ox-mangiferin (*Ps*G3Ox-Mang) complex structures. The cavity contains the residues ^{125}AAHW^{128}, the substrate loop (^{346}ASPVPLADD^{354}), and the insertion-1 segment (60-93).

Structure	Residues	Volume (Å^3)	Area (Å^2)	Depth (Å)	Lenght (Å)
*Ps*G3Ox (Tunnel)	V80, V86, G89, E90, R92, R94, V114, T129, Q297, P348, V349, F368, F431, L433, P434, A437, S438, L439, H440, N484	-	-	-	25
*Ps*G3Ox (Cavity)	Q67, R94, P95, H127, W128, T129, L344, D345, S347, P348, V349, P350	117.7	264.0	11.3	-
*Ps*G3Ox-Glc (Cavity)	Q67, R94, P95, H127, W128, T129, Q297, Q340, L344, F368	187.5	298.8	14.1	-
*Ps*G3Ox-Mang (Cavity)	Q67, R94, P95, T97, S118, H127, W128, T129, A131, Q297, Q340, F368, A437, S438	282.3	503.2	15.9	-

Supplementary Table 6. Apparent steady-state kinetic parameters for D-Glc and mangiferin of *Ps*G3Ox variants. The catalytic parameters for D-Glc were estimated using the HRP-AAP/DCHBS coupled assay in a reaction mixture containing 0-1.25 M D-Glc, 0.1 mM of AAP, 1 mM of DCHBS and 8 U ml^{-1} of HRP, whereas reactions with mangiferin were monitored by oxygen consumption in an Oxygraph. The data is represented as mean ± SD of the independent experiments (n=3). All reactions were performed in 100 mM sodium phosphate buffer at pH 7.5 at 37 ºC. The kinetic parameters were determined by fitting the data directly on the Michaelis-Menten equation using OriginLab. Source data are provided as a Source Data file.

	**D-Glucose**			**Mangiferin**		
**Enzyme**	k_{cat} (s^{-1})	K_m(M)	k_{cat}/ K_m($M^{-1}s^{-1}$)	k_{cat}(s^{-1})	K_m(M)	k_{cat}/ K_m($M^{-1}s^{-1}$)
Wild-type	0.19 ± 0.03	0.5 ± 0.1	0.45 ± 0.09	8.1 ± 1.7	(0.5 ± 0.1) ×10^{-3}	(19.0 ± 3.0) ×10^{3}
A125S	0.14 ± 0.02	0.6 ± 0.1	0.24 ± 0.03	ND	-	-
A126T	0.09 ± 0.03	0.6 ± 0.1	0.15 ± 0.06	ND	-	-
A125S-A126T	nd	-	-	nd	-	-
H440A	nd	-	-	nd	-	-
N484A	nd	-	-	nd	-	-
N120V	nd	-	-	ND	-	-
Q340A	(0.30 ± 0.04) ×10^{-2}	0.5 ± 0.1	(0.70 ± 0.04) ×10^{-2}	0.6 ± 0.1	(1.2 ± 0.1) ×10^{-3}	(0.4 ± 0.1) ×10^{3}
Q297A	0.04 ± 0.01	0.7 ± 0.1	0.08 ± 0.01	2.2 ± 0.5	(0.7 ± 0.2) ×10^{-3}	(2.7 ± 0.1) ×10^{3}
K55A	0.11 ± 0.02	0.4 ± 0.1	0.31 ± 0.06	9.5 ± 2.7	(1.5 ± 0.4) ×10^{-3}	(8.7 ± 0.8) ×10^{3}
R94A	0.20 ± 0.04	0.6 ± 0.2	0.36 ± 0.05	0.7 ± 0.1	(1.8 ± 0.3) ×10^{-3}	(0.4 ± 0.1) ×10^{3}
T129A	0.09 ± 0.01	0.2 ± 0.02	0.53 ± 0.03	0.5 ± 0.1	(0.7 ± 0.2) ×10^{-3}	(0.7 ± 0.1) ×10^{3}

ND-not determined; nd-not detected

Supplementary Table 7. Atomic displacement parameters (<*a.d.p.*>) values (Å^2) of residues in substrate loops of POxs with known crystal structure. *Ps*G3Ox (346-354) from *P. siccitolerans*; *Mt*CarA (350-358) from *M. trichothecenolyticum; Tm*P2Ox (452-460) from *T. multicolor*; *Ps*P2Ox (452-460) from *Peniophora sp. SG* and *Pc*P2Ox (458-466) from *P. chrysosporium*. The <*a.d.p.*> values are shown in parentheses.

**Bacterial**		**Fungal**		
*Ps*G3Ox (PDB 7QF8)	*Mt*CarA (PDB 7DVE)	*Tm*P2Ox (PDB 2IGK)	*Ps*P2Ox (PDB 1TZL)	*Pc*P2Ox (PDB 4MIF)
A346 (50.0)	A350 (47.5)	D452 (38.6)	D452 (37.4)	D458 (67.8)
S347 (49.4)	S351 (49.7)	A453 (41.7)	A453 (41.9)	A459 (57.8)
P348 (48.7)	P352 (50.3)	F454 (43.8)	F454 (47.7)	F460 (62.8)
V349 (53.9)	V353 (54.0)	S455 (47.3)	S455 (48.1)	S461 (63.0)
P350 (58.1)	P354 (60.7)	Y456 (50.5)	Y456 (48.3)	Y462 (54.5)
L351 (64.4)	L355 (57.8)	G457 (48.6)	G457 (49.0)	G463 (55.9)
A352 (72.7)	A356 (61.1)	A458 (48.7)	A458 (48.6)	A464 (65.1)
D353 (84.0)	D357 (64.4)	V459 (46.6)	V459 (46.6)	V465 (71.4)
D354 (88.2)	D358 (57.9)	Q460 (38.3)	Q460 (40.5)	A466 (52.1)

Supplementary Table 8. Solvent accessible surface area (ASA) values of residues present in substrate loops of POxs with known crystal structure. *Ps*G3Ox (346-354) from *P. siccitolerans*; *Mt*CarA (350-358) from *M. trichothecenolyticum; Tm*P2Ox (452-460) from *T. multicolor*; *Ps*P2Ox (452-460) from *Peniophora sp. SG* and *Pc*P2Ox (458-466) from *P. chrysosporium*. The ASA values in % are shown in parenthesis.

**Bacterial**		**Fungal**		
*Ps*G3Ox (PDB 7QF8)	*Mt*CarA (PDB 7DVE)	*Tm*P2Ox (PDB 2IGK)	*Ps*P2Ox (PDB 1TZL)	*Pc*P2Ox (PDB 4MIF)
A346 (4)	A350 (2)	D452 (13)	D452 (8)	D458 (20)
S347 (14)	S351 (15)	A453 (10)	A453 (8)	A459 (17)
P348 (11)	P352 (11)	F454 (23)	F454 (20)	F460 (25)
V349 (7)	V353 (13)	S455 (35)	S455 (33)	S461 (74)
P350 (28)	P354 (73)	Y456 (29)	Y456 (28)	Y462 (13)
L351 (9)	L355 (24)	G457 (55)	G457 (51)	G463 (53)
A352 (19)	A356 (20)	A458 (72)	A458 (57)	A464 (84)
D353 (69)	D357 (70)	V459 (59)	V459 (65)	V465 (55)
D354 (37)	D358 (33)	Q460 (43)	Q460 (13)	A466 (5)

Supplementary Table 9. Solvent accessible surface area (ASA) values of amino acid residues limiting the cavities in *Ps*G3Ox wild-type, *Ps*G3Ox-glucose (*Ps*G3Ox-Glc), and *Ps*G3Ox-mangiferin (*Ps*G3Ox-Mang) complex structures.

Residues	*Ps*G3Ox	*Ps*G3Ox-Glc	*Ps*G3Ox-Mang
Q67	19	21	21
R94	15	19	28
P95	20	49	51
T97	1	3	3
S118	0	0	0
H127	9	7	6
W128	1	1	0
T129	8	17	15
A131	3	2	1
Q297	4	7	6
Q340	1	2	4
L344	20	66	28
D345	36	-	85
S347	14	-	37
P348	11	-	55
V349	7	-	39
P350	28	-	93
F368	1	1	1
A437	6	3	5
S438	4	0	1

Supplementary Table 10. Binding free energies (*Δ*G, in kcal/mol) calculated for D-Glc and Mang by the Molecular Mechanics/Poisson–Boltzmann Surface Area (MM-PBSA) and the Molecular Mechanics/Generalised Born Surface Area (MM-GBSA) methods. Relative binding free energies (*ΔΔ*G, in kcal/mol) are calculated using only the averaged values from the simulations that are structurally consistent with catalysis (see also Supplementary Figure 30).

**Trajectory number**	ΔG_{GB}(D-Glc) (kcal/mol)	ΔG_{GB}(Mang) (kcal/mol)	ΔG_{PB}(D-Glc) (kcal/mol)	ΔG_{PB}(Mang) (kcal/mol)
1	-41.0	-39.9	-8.2	-6.7
2	-65.8	-40.6	-26.8	-7.3
**3**	-36.9	-44.2	-7.5	-13.3
**4**	-38.2	-26.8	-14.0	5.4
5	-30.1	-38.1	-8.4	-5.7
6	-39.4	-40.8	-11.0	-10.9
7	-31.9	-42.5	-3.1	-12.7
8	-28.1	-27.8	2.0	4.5
**9**	-37.8	-37.2	-5.1	-8.8
10	-32.9	-28.0	-5.9	2.9
**Average all**	**-38.2**	**-36.6**	**-8.8**	**-5.3**
Average catalytic	-36.9	-41.2	-7.8	-11.4
*ΔΔ*G (Mang vs D-Glc) (kcal/mol)	-4.3		-3.6	

Supplementary Table 11. Bacterial strains, plasmids, and primers used in the construction of *Ps*G3Ox variants. F indicates forward primers, and R reverse primers.

Strains, Plasmids, or Primers	Genotype, property, or sequence	Reference or source
***E. coli* Strains**		
DH5**α**	F^{-} φ80*lac*ZΔM15 Δ(*lac*ZYA *arg*F)U169 *rec*A1 *end*A1 *hsd*R17(r_K^-, m_K^+) *pho*A *sup*E44 λ^{-} *thi*-1 *gyr*A96 *rel*A1	Novagen
Rosetta(DE3) pLysS	F^{-} ompT hsdSB(rB- mB-) gal dcm (DE3) pLysSRARE (CamR)	Novagen
**Plasmids**		
pET15b	Cloning vector with a T7 promoter with a N-terminal T7tag and C-terminal 6xHis tag; amp^{r}	Novagen
pSM1	pET15b with *psg3ox* gene cloned into *Nde*I and *XhoI* sites	(Mendes et al., 2016)
pDS1	pET15b with *psg3ox* gene cloned into *Nde*I and *XhoI* sites. The A125S mutation was introduced by site-directed mutagenesis	This work
pDS2	pET15b with *psg3ox* gene cloned into *Nde*I and *XhoI* sites. The A126T mutation was introduced by site-directed mutagenesis	This work
pDS3	pET15b with *psg3ox* gene cloned into *Nde*I and *XhoI* sites. The A125S and A126T mutations were introduced by site-directed mutagenesis	This work
pDS13	pET15b with *psg3ox* gene cloned into *Nde*I and *XhoI* sites. The H440A mutation was introduced by site-directed mutagenesis	This work
pDS14	pET15b with *psg3ox* gene cloned into *Nde*I and *XhoI* sites. The N484A mutation was introduced by site-directed mutagenesis	This work
pAT16	pET15b with *psg3ox* gene cloned into *Nde*I and *XhoI* sites. The N120V mutation was introduced by site-directed mutagenesis	This work
pAT27	pET15b with *psg3ox* gene cloned into *Nde*I and *XhoI* sites containing the deletion of the residues 345-359 (Δloop(345-359)) introduced by site-directed mutagenesis	This work
pAT28	pET15b with *psg3ox* gene cloned into *Nde*I and *XhoI* sites containing the deletion of the residues 73-93 (Δinsertion-1) introduced by site-directed mutagenesis	This work
pAT29	pET15b with *psg3ox* gene cloned into *Nde*I and *XhoI* sites. The R94A mutation was introduced by site-directed mutagenesis	This work
pAT31	pET15b with *psg3ox* gene cloned into *Nde*I and *XhoI* sites. The Q297A mutation was introduced by site-directed mutagenesis	This work

pAT33	pET15b with *psg3ox* gene cloned into *Nde*I and *XhoI* sites. The K55A mutation was introduced by site-directed mutagenesis	This work
pAT35	pET15b with *psg3ox* gene cloned into *Nde*I and *XhoI* sites. The Q340A mutation was introduced by site-directed mutagenesis	This work
pAT39	pET15b with *psg3ox* gene cloned into *Nde*I and *XhoI* sites. The T129A mutation was introduced by site-directed mutagenesis	This work
**Primers**		
PsG3Ox-A125S F	5'-GCAACGTGGGCGGGATGTCGGCCCACTGGACCGCCGCCTG-3'	This work
PsG3Ox -A125S R	5'-CAGGCGGCGGTCCAGTGGGCCGACATCCCGCCCACGTTGC-3'	This work
PsG3Ox -A126T F	5'-CGTGGGCGGGATGGCCACCCACTGGACCGCCGCCTGC-3'	This work
PsG3Ox -A126T R	5'-GCAGGCGGCGGTCCAGTGGGTGGCCATCCCGCCCACG-3'	This work
PsG3Ox -A125S/A126T F	5'-GCAACGTGGGCGGGATGTCCACCCACTGGACCGCCGCCTGC-3'	This work
PsG3Ox -A125S/A126T R	5'-GCAGGCGGCGGTCCAGTGGGTGGACATCCCGCCCACGTTGC-3'	This work
PsG3Ox -H440A F	5'-GCCGGGTGCGTCGCTGGCCTACCAGGGCACCA-3'	This work
PsG3Ox -H440A R	5'-TGGTGCCCTGGTAGGCCAGCGACGCACCCGGC-3'	This work
PsG3Ox -N484A F	5'-CCACCGCTACAGCATGCGCCCCCACCCTGACGT-3'	This work
PsG3Ox -N484A R	5'-ACGTCAGGGTGGGGGCGCATGCTGTAGCGGTGG-3'	This work
PsG3Ox -N120V F	5'- CCATGTCCAGCGTCGTGGGCGGGATGG -3'	This work
PsG3Ox -N120V R	5'- CCATCCCGCCCACGACGCTGGACATGG -3'	This work
PsG3Ox -del345-359 F	5'(Phosphorylated)-GAGCTGCATGATCTGGCCGTGGAAGGGCGC-3'	This work
PsG3Ox -del345-359 R	5'(Phosphorylated)-GGCTCCATCGTGGGGCTGGGCCTGTTCTGC-3'	This work
PsG3Ox -del73-93 F	5'(Phosphorylated)-CGCCCTGGAACTTACCTGCTGCAGGACGG-3'	This work
PsG3Ox -del73-93 R	5'(Phosphorylated)-ACCCTCCGACGCCCGCTGGGCG-3'	This work
PsG3Ox -R94A F	5'-CGAACGCCGTGCGGCCCCTGGAACTTACC-3'	This work
PsG3Ox – R94A R	5'-GGTAAGTTCCAGGGGCCGCACGGCGTTCG-3'	This work
PsG3Ox -Q297A F	5'-CCTGAACGACCAGGCGGCCGTGGTGTTCGCG-3'	This work
PsG3Ox – Q297A R	5'-CGCGAACACCACGGCCGCCTGGTCGTTCAGG-3'	This work
PsG3Ox -K55A F	5'-CGCGCACGTCGCGAACATCGAGGACC-3'	This work
PsG3Ox – K55A R	5'-GGTCCTCGATGTTCGCGACGTGCGCG-3'	This work
PsG3Ox -Q340A F	5'-CCTTCCACGGCGCGATCATGCAGC-3'	This work

PsG3Ox – Q340A R	5’-GCTGCATGATCGCGCCGTGGAAGG-3’	This work
PsG3Ox -T129A F	5’-CGCCCACTGGGCTGCCGCCTGC-3’	This work
PsG3Ox – T129A R	5’-GCAGGCGGCAGCCCAGTGGGCG-3’	This work

Supplementary Table 12. Additional details of the systems' setup for the molecular dynamics simulations. For each model built to run MD, the dimensions of the simulation box (in Å), the total number of atoms, the number of water molecules and a comment on the salt concentration are given.

Systems setup	Simulation box dimensions (x,y,z)(Å)			Total number of atoms	Number of water molecules	Salt concentration
**Model I (cMD)**	90	85	80	66754	19718	Ions (Na^+) added for charge neutralization only.
**Model II (cMD)**	74	92	74	57187	16521	Ions (Na^+) added for charge neutralization only.
**Model III (cMD)**	72	96	93	700632	21012	Ions (Na^+) added for charge neutralization only.
**Model IV (cMD)**	90	85	83	71112	21143	Ions (Na^+) added for charge neutralization only.
**Model I (GaMD)**	79	96	76	46660	13020	Ions (Na^+) added for charge neutralization only.
**Model II (GaMD)**	75	94	75	41971	11449	Ions (Na^+) added for charge neutralization only.
**Model III (GaMD)**	82	95	75	46794	13066	Ions (Na^+) added for charge neutralization only.
**Model III* (GaMD)**	82	95	75	46794	13066	Ions (Na^+) added for charge neutralization only.
**Glc (cMD, 100ns)**	79	82	72	47158	13178	Ions (Na^+) added for charge neutralization only.
**Mang (cMD, 100ns)**	82	81	75	50178	14186	Ions (Na^+) added for charge neutralization only.

4. Supplementary References

1. Kocourek, J., Ticha, M. & Kostir, J. The use of diphenylamine-alanine-phosphoric acid reagent in the detection and differentiation of monosaccharides and their derivatives on paper chromatograms. *J Chromatogr* **24**, 117-24 (1966).
2. Kujawa, M. et al. Structural basis for substrate binding and regioselective oxidation of monosaccharides at C3 by pyranose 2-oxidase. *J Biol Chem* **281**, 35104-15 (2006).
3. Spadiut, O., Tan, T.C., Pisanelli, I., Haltrich, D. & Divne, C. Importance of the gating segment in the substrate-recognition loop of pyranose 2-oxidase. *FEBS J* **277**, 2892-909 (2010).
4. Tan, T.C., Haltrich, D. & Divne, C. Regioselective control of beta-d-glucose oxidation by pyranose 2-oxidase is intimately coupled to conformational degeneracy. *J Mol Biol* **409**, 588-600 (2011).
5. Krieger, E. & Vriend, G. YASARA View-molecular graphics for all devices-from smartphones to workstations. *Bioinformatics* **30**, 2981-2982 (2014).
6. Zhang, K.K. et al. Identification and characterization of a novel bacterial pyranose 2-oxidase from the lignocellulolytic bacterium *Pantoea ananatis* Sd-1. *Biotechnol Lett* **40**, 871-880 (2018).
7. Herzog, P.L. et al. Versatile oxidase and dehydrogenase activities of bacterial pyranose 2-oxidase facilitate redox cycling with manganese peroxidase in vitro. *Appl Envriron Microbiol* **85**, 1-15 (2019).
8. Kumano, T. et al. FAD-dependent C-glycoside-metabolizing enzymes in microorganisms: Screening, characterization, and crystal structure analysis. *Proc Nat Aca Sci USA* **118**, e2106580118 (2021).
9. Kostelac, A. et al. Biochemical Characterization of Pyranose Oxidase from *Streptomyces canus*-Towards a Better Understanding of Pyranose Oxidase Homologues in Bacteria. *Int J Mol Sci* **23**, 13595 (2022).
10. Schafer, A., Bieg, S., Huwig, A., Kohring, G. & Giffhorn, F. Purification by Immunoaffinity Chromatography, Characterization, and Structural Analysis of a Thermostable Pyranose Oxidase from the White Rot Fungus *Phlebiopsis gigantea*. *Appl Environ Microbiol* **62**, 2586-92 (1996).
11. Leitner, C., Volc, J. & Haltrich, D. Purification and characterization of pyranose oxidase from the white rot fungus *Trametes multicolor*. *Appl Environ Microbiol* **67**, 3636-44 (2001).
12. Takakura, Y. & Kuwata, S. Purification, characterization, and molecular cloning of a pyranose oxidase from the fruit body of the basidiomycete, *Tricholoma matsutake*. *Biosci Biotechnol Biochem* **67**, 2598-607 (2003).
13. Bastian, S., Rekowski, M.J., Witte, K., Heckmann-Pohl, D.M. & Giffhorn, F. Engineering of pyranose 2-oxidase from *Peniophora gigantea* towards improved thermostability and catalytic efficiency. *Appl Microbiol Biotechnol* **67**, 654-63 (2005).
14. Bannwarth, M., Heckmann-Pohl, D., Bastian, S., Giffhorn, F. & Schulz, G.E. Reaction geometry and thermostable variant of pyranose 2-oxidase from the white-rot fungus *Peniophora* sp. *Biochemistry* **45**, 6587-95 (2006).
15. Pisanelli, I. et al. Pyranose 2-oxidase from Phanerochaete chrysosporium--expression in E. coli and biochemical characterization. *J Biotechnol* **142**, 97-106 (2009).
16. Salaheddin, C. et al. Characterisation of recombinant pyranose oxidase from the cultivated mycorrhizal basidiomycete *Lyophyllum shimeji* (hon-shimeji). *Microb Cell Fact* **9**, 57 (2010).
17. Pisanelli, I. et al. Heterologous expression and biochemical characterization of novel pyranose 2-oxidases from the ascomycetes *Aspergillus nidulans* and *Aspergillus oryzae*. *Appl Microbiol Biotechnol* **93**, 1157-66 (2012).

18. Ai, M.Q., Wang, F.F., Zhang, Y.Z. & Huang, F. Purification of pyranose oxidase from the white rot fungus *Irpex lacteus* and its cooperation with laccase in lignin degradation. *Proc Biochem* **49**, 2191-2198 (2014).
