# Supplementary material for: Mechanistic insights into glycoside 3-oxidases involved in C-glycoside metabolism in soil microorganisms
Source: Nat Commun. 2023 Nov 14;14:7289. doi: 10.1038/s41467-023-42000-3 (PMC10646112; doi:10.1038/s41467-023-42000-3)
Supplement: Supplementary file 2 — Reporting Summary [file 41467_2023_42000_MOESM2_ESM.pdf]

nature portfolio

Corresponding author(s): *Double-anonymous peer review submissions: write DAPR and your manuscript number here instead of author names.*

Last updated by author(s): *YYYY-MM-DD*

Reporting Summary

Nature Portfolio wishes to improve the reproducibility of the work that we publish. This form provides structure for consistency and transparency in reporting. For further information on Nature Portfolio policies, see our Editorial Policies and the Editorial Policy Checklist.

Statistics

For all statistical analyses, confirm that the following items are present in the figure legend, table legend, main text, or Methods section.

n/a	Confirmed	
☐	☒	The exact sample size (*n*) for each experimental group/condition, given as a discrete number and unit of measurement
☐	☒	A statement on whether measurements were taken from distinct samples or whether the same sample was measured repeatedly
☒	☐	The statistical test(s) used AND whether they are one- or two-sided<br>*Only common tests should be described solely by name; describe more complex techniques in the Methods section.*
☒	☐	A description of all covariates tested
☒	☐	A description of any assumptions or corrections, such as tests of normality and adjustment for multiple comparisons
☐	☒	A full description of the statistical parameters including central tendency (e.g. means) or other basic estimates (e.g. regression coefficient) AND variation (e.g. standard deviation) or associated estimates of uncertainty (e.g. confidence intervals)
☒	☐	For null hypothesis testing, the test statistic (e.g. *F*, *t*, *r*) with confidence intervals, effect sizes, degrees of freedom and *P* value noted<br>*Give P values as exact values whenever suitable.*
☒	☐	For Bayesian analysis, information on the choice of priors and Markov chain Monte Carlo settings
☒	☐	For hierarchical and complex designs, identification of the appropriate level for tests and full reporting of outcomes
☒	☐	Estimates of effect sizes (e.g. Cohen's *d*, Pearson's *r*), indicating how they were calculated

Our web collection on statistics for biologists contains articles on many of the points above.

Software and code

Policy information about availability of computer code

Data collection	Steady-state kinetic data were collected using BioTek Gen5 software (version 1.11) for spectrophotometric or Oxytrace+ for Oxygraph assays. Transient-state kinetics were collected in kinetic studio. The X-ray crystallographic data were collected in beamlines 1023-2 and 1030A-1 at the European Synchrotron Radiation Facility (ESRF, Grenoble, France) and in the XALOC beamline at ALBA (Barcelona, Spain). Conventional molecular dynamics simulations and protein-ligand docking calculations were run with the commercial YASARA (version 19.9.12) program, whereas for the Gaussian accelerated molecular dynamics (GaMD) simulations, the AMBER20 commercial program was used; in both cases, no code modifications were performed.
Data analysis	Steady-state kinetics were analysed using Origin2018. Transient-state kinetics were analysed using KineticStudio and Solver ad tool of Microsoft Excel. The phylogenetic tree was constructed using MEGA 11 software. The NMR spectra were analysed using Bruker topspin 3.2 software. Crystallographic software used: Global Phasing Limited autoPROC (October 20, 2021), MORDA 33 (June 29, 2020), PHENIX (version 1.19.2-4158), COOT (version 0.8.9.2), CCP4 (version 7.0), BUSTER (version 2.10), MOLPROBITY (version 4.5.2), MODELLER (version 9.12-1-amd64), MOLE (version 2.13.9.6), DogSitesScorer and Alphafold V2, Rosetta (version 2020.08.61146), PyMOL (version 1.8-x). Simulations were analyzed using in-house scripts, available on GitHub (https://github.com/insilichem/utils_PsG3Ox/)

For manuscripts utilizing custom algorithms or software that are central to the research but not yet described in published literature, software must be made available to editors and reviewers. We strongly encourage code deposition in a community repository (e.g. GitHub). See the Nature Portfolio guidelines for submitting code & software for further information.

Data

Policy information about availability of data

All manuscripts must include a data availability statement. This statement should provide the following information, where applicable:

- Accession codes, unique identifiers, or web links for publicly available datasets
- A description of any restrictions on data availability
- For clinical datasets or third party data, please ensure that the statement adheres to our policy

The structural coordinates generated in this study have been deposited in the Protein Data Bank under accession codes: 7QF8 [https://doi.org/10.2210/pdb7qf8/pdb], 7QFD [https://doi.org/10.2210/pdb7qfd/pdb], and 7QVA [https://doi.org/10.2210/pdb7qva/pdb]. The biochemical data generated in this study are provided in the Supplementary Information/Source Data file. The structural data used in this study are available in the Protein Data Bank database under accession code 7DVE [https://doi.org/10.2210/pdb7dve/pdb], 1TT0 [https://doi.org/10.2210/pdb1TT0/pdb], 4MIF [https://doi.org/10.2210/pdb4MIF/pdb], 2IGK [https://doi.org/10.2210/pdb2IGK/pdb], 3OD1 [https://doi.org/10.2210/pdb3OD1/pdb] and 1TZL [https://doi.org/10.2210/pdb1TZL/pdb]. Source data are provided with this paper. The molecular dynamics input files, as well as the initial and final coordinates of each simulations run are provided in a Supplementary Information zipped folder.

Research involving human participants, their data, or biological material

Policy information about studies with human participants or human data. See also policy information about sex, gender (identity/presentation), and sexual orientation and race, ethnicity and racism.

Reporting on sex and gender	This does not apply to this study
Reporting on race, ethnicity, or other socially relevant groupings	This does not apply to this study
Population characteristics	This does not apply to this study
Recruitment	This does not apply to this study
Ethics oversight	This does not apply to this study

Note that full information on the approval of the study protocol must also be provided in the manuscript.

Field-specific reporting

Please select the one below that is the best fit for your research. If you are not sure, read the appropriate sections before making your selection.

☒ Life sciences ☐ Behavioural & social sciences ☐ Ecological, evolutionary & environmental sciences

For a reference copy of the document with all sections, see nature.com/documents/nr-reporting-summary-flat.pdf

Life sciences study design

All studies must disclose on these points even when the disclosure is negative.

Sample size	For all statistical experiments, including kinetic analysis, values were determined with at least n = 3, as it is common practice in the field.
Data exclusions	No data were excluded.
Replication	All experiments were performed independently and successfully replicated at least three times. Furthermore, the reproducibility of the assays was confirmed by including appropriate positive and negative controls
Randomization	This is not relevant as we have studied applying biochemical and structural methodologies one defined enzyme; the products, catalysed reactions and structures were unknown during these assays.
Blinding	This is irrelevant to this study because we used enzyme variants for in vitro reactions. The products, catalyzed reactions, and structures were unknown during these assays.

Reporting for specific materials, systems and methods

We require information from authors about some types of materials, experimental systems and methods used in many studies. Here, indicate whether each material, system or method listed is relevant to your study. If you are not sure if a list item applies to your research, read the appropriate section before selecting a response.

Materials & experimental systems

n/a	Involved in the study	
☒	☐	Antibodies
☒	☐	Eukaryotic cell lines
☒	☐	Palaeontology and archaeology
☒	☐	Animals and other organisms
☒	☐	Clinical data
☒	☐	Dual use research of concern
☒	☐	Plants

Methods

n/a	Involved in the study	
☒	☐	ChIP-seq
☒	☐	Flow cytometry
☒	☐	MRI-based neuroimaging
