# Supplementary material for: Mechanistic insights into glycoside 3-oxidases involved in C-glycoside metabolism in soil microorganisms
Source: Nat Commun. 2023 Nov 14;14:7289. doi: 10.1038/s41467-023-42000-3 (PMC10646112; doi:10.1038/s41467-023-42000-3)
Supplement: Supplementary file 4 — Description of Additional Supplementary Files [file 41467_2023_42000_MOESM4_ESM.pdf]

Title: Supplementary Data 1.
Description: Input files, initial and final coordinates of all the molecular dynamics simulations performed in this study: the long conventional MDs (cMDs) of Model I - IV, the Gaussian Accelerated MDs (GAMDs) of Model I-III, III*, and the short cMDs used in the MMPBSA calculations of the binding free energy for glucose and mangiferin substrates.
